# *Notch1* deletion reduces arrhythmogenic remodeling after myocardial infarction

**DOI:** 10.1016/j.hrthm.2026.03.1957

**Published:** 2026-04-07

**Authors:** Emanuele Pizzo, Daniel O. Cervantes, Giulia Piccinini, Belinda Nyantakyi, Maria T. Ochoa, Michael Y. Sun, Andrea Filardo, Harshada Ketkar, Sudhir Jain, Jason T. Jacobson, Malik Bisserier, Marcello Rota

**Affiliations:** 1 Department of Cell and Molecular Physiology, New York Medical College, Valhalla, New York,; 2 Department of Pathology, Microbiology and Immunology, New York Medical College, Valhalla, New York,; 3 Division of Cardiology, Department of Medicine, Westchester Medical Center, Valhalla, New York,; 4 Department of Medicine, New York Medical College, Valhalla, New York.

**Keywords:** Notch signaling, Post-MI remodeling, Myocytes, Transient Kv current, Arrhythmias

## Abstract

**BACKGROUND:**

Myocardial infarction (MI) induces re-expression of fetal genes, including *Notch1*, which can modify the electrophysiological properties of cardiomyocytes.

**OBJECTIVE:**

This study aimed to evaluate the role of Notch1 signaling in the electrical remodeling of the injured heart.

**METHODS:**

Electrophysiological consequences of *Notch1* gene deletion were evaluated in male and female conditional knockout (cKO) mice, with respect to corresponding wild-type (WT) animals. Mice were studied both in the naïve state and after MI.

**RESULTS:**

Electrocardiographic recordings showed no arrhythmias in WT and cKO mice in the naïve state. However, 1 day after MI, 69% of WT mice exhibited ectopic beats compared with 31% of cKO animals, who also presented lower arrhythmia burden. To examine the effects of Notch1 loss on myocardial repolarization, monophasic action potentials (MAPs) were recorded in perfused hearts. In the myocardium of WT mice, MAP duration was prolonged after MI, whereas this effect was attenuated in cKO hearts, resulting in shorter MAP duration in cKO hearts with respect to WT organs. To identify the ionic basis for this difference, patch-clamp recordings were performed on isolated left ventricular myocytes. In the naïve state, transient outward voltage-gated potassium currents were comparable between WT and cKO cells. However, ~2 weeks after MI, WT myocytes showed a significant reduction of total voltage-gated potassium currents, whereas cKO myocytes maintained levels similar to baseline.

**CONCLUSION:**

*Notch1* deficiency mitigates the electrophysiological remodeling of cardiomyocytes and is coupled with reduced arrhythmia susceptibility of the heart after MI.

Rapidly activating transient outward voltage-gated potassium (K^+^) (Kv) currents play a major role in the repolarization phase of the action potential (AP) in adult cardiomyocytes.^1,2^ Transient Kv currents are developmentally regulated,^3^ and their expression is diminished under pathologic conditions, contributing to the characteristic prolongation of the AP observed in cells from postinfarcted, hypertrophied, failing, or aging hearts.^4–6^ The loss of Kv currents and subsequent AP prolongation significantly affect the electromechanical behavior of cardiomyocytes and the propensity of the heart to develop arrhythmias.^2,5,7,8^ However, the mechanisms underlying the modulation of Kv current under diseased states remain incompletely understood.

Notch1 receptor signaling, which is active during cardiac development, becomes progressively silenced after birth but can be transiently restored in the adult myocardium under pathologic conditions,^9–12^ yet the functional implications of Notch1 re-expression in the diseased heart are not fully defined. Notably, activation of Notch1 signaling in adult cardiomyocytes represses Kv currents leading to prolongation of the AP,^12,13^ whereas inhibition or silencing of Notch signaling in developing myocytes enhances Kv currents.^13,14^ These observations suggest that Notch signaling may serve as a common regulatory system governing the electrical properties of cardiomyocytes during both development and diseased conditions.

In this study, we investigate the impact of *Notch1* gene deletion on the electrophysiological and functional properties of the heart after myocardial infarction (MI). Using genetically engineered mice and isolated cardiomyocytes, we provide evidence that Notch1 signaling contributes to the electrical remodeling and arrhythmogenic propensity of the injured heart.

## Methods

All data, materials, and methods of this study are available from the corresponding author upon reasonable request.

The research reported in this article adhered to Animal Research: Reporting of In Vivo Experiments guidelines and the Guide for the Care and Use of Laboratory Animals. Animal housing and experiments were approved by the local animal care committee (Institutional Animal Care and Use Committee) of New York Medical College.

For this investigation, male and female mice with cardiacrestricted, inducible deletion of the *Notch1* gene (conditional knockout [cKO]) and corresponding wild-type (WT) mice were studied in the naïve state and after MI. At the time of data collection, mouse age ranged from 3 to 8 months. Some of the animals used here were enrolled in a separate project addressing the consequences of MI on heart rate variability.^15^

To obtain mice with conditional and inducible deletion of the *Notch1* gene in myocytes, a *Notch1*^*flox/flox*^/αMHC-MerCreMer (cKO) mouse line was obtained by intercrossing *Notch1* ^*flox/flox*^ animals (B6.129X1-Notch1^tm2Rko^/Grid, Jackson Laboratory, stock no. 7181)^16,17^ with *B6.FVB(129)-A1cf*^Tg(Myh6-cre/Esr1*)1Jmk^*/J* mice expressing tamoxifen-inducible Cre recombinase under the α-myosin heavy chain promoter (αMHC-MerCreMer, Jackson Laboratory, stock no. 005657).^13,18^ Both mouse lines were backcrossed onto the C57BL/6J congeneric background by the vendor. The inducible, cardiac-restricted model of *Notch1* deletion (cKO) was used to circumvent the lethality of offspring associated with the complete loss of the *Notch1* gene.^17^ Single transgenic mice homozygous for the MerCreMer construct were treated with tamoxifen and used as controls.^13,15,19^

Cre recombinase expression was induced by administration of tamoxifen (MilliporeSigma). Tamoxifen was dissolved in 10% ethanol and 90% peanut oil (MilliporeSigma) and administered to mice with 4 injections (30 mg/kg of body weight/injection, intraperitoneally [i.p.]) on different days (no more than 1 injection/d), over a period of 4–8 days.^15,19^ The survival pattern after tamoxifen treatment was comparable in WT and cKO mice (Supplemental Figure 1A) and consistent with previous studies.^19^ Mice were enrolled in the study at 3 or more weeks after the beginning of tamoxifen treatment. By Western blotting, expression of Notch1 in tamoxifen-treated mice was reduced in the left ventricular (LV) myocardium of cKO naïve or infarcted mice, with respect to the LV myocardium of corresponding WT animals (Supplemental Figure 1B–1D). Unless otherwise specified, collected data were disaggregated by sex.

### MI

MI was performed under sterile conditions via thoracotomy and coronary artery ligation.^15,20,21^ Under isoflurane anesthesia (~1.5%), the animal was intubated and ventilated continuously during the surgical procedure. A local anesthetic agent (lidocaine, ~4 mg/kg) was injected subcutaneously in the incision site before and after the surgical procedure. The thorax was opened via the third intercostal space, and the atrial appendage was elevated. The left coronary artery was located, and a suture (6–0 Silk DR12 Black 18” Braid, Henry Schein) was inserted around the vessel near the origin and the artery was occluded. The chest was closed with a suture, and the pneumothorax was reduced by negative pressure. Skin incision was closed using a 9 mm wound clip (Fine Science Tools). To reduce postoperative pain after the procedure, buprenorphine hydrochloride (Buprenex), 0.5–1 mg/kg body weight, was injected i.p. every 12 hours for a period of 48 hours after surgery.

### Electrocardiographic recording in the conscious state

To record electrocardiograms (ECGs) in conscious animals, an ECG-tunnel device (Emka Technologies) was used.^22^ Animals were placed in a tunnel and ECGs were recorded for a period of 10 minutes. Electrical signals were amplified using a 12-lead ECG amplifier (DSI, Ponemah), digitized using a 160 kHz analog-to-digital (A/D) converter (DI-1120 HS, Dataq), and recorded with WinDaq software (Dataq). The bipolar leads I, II, and III, and the unipolar lead aVL were collected. Electrical signals were evaluated offline using LabChart 8 for the analysis of the occurrence of rhythm disturbances.^22–24^

#### Analysis of electrical stability

The occurrence of arrhythmic events was evaluated in ECGs collected in the conscious state, using LabChart 8 software.^15,19,22^ Quantifications were conducted to establish the fraction of mice presenting isolated or multiple ventricular ectopic beats, identified as beats characterized by atrioventricular dissociation, prematurity of the QRS complex, and QRS morphology differing from that of the QRS in sinus rhythm, including wider QRS interval duration. Mice presenting 10 or more isolated ventricular ectopic beats over the 10-minute period of acquisition were referred to as animals with recurrent ventricular ectopic beats. Ventricular tachycardia (VT) was identified as 3 or more consecutive ectopic beats.^15,23^ Premature junctional complexes were identified as ectopic beats characterized by atrioventricular dissociation and prematurity of the QRS complex, but QRS morphology and duration were comparable with QRS properties of sinus rhythm. These features are consistent with premature beats arising from an ectopic focus within the atrioventricular junction. A cumulative ventricular arrhythmia score was assigned to each mouse based on the occurrence of 1 or multiple forms of ventricular ectopy. Arrhythmic events were scored using a system adapted previously,^24^ according to the following parameters: 0, absence of ventricular ectopic beats; 1, isolated ventricular ectopic beats (<10 in 10 minutes); 2, recurrent ventricular ectopic beats (≥10 in 10 minutes); 3, individual or multiple couplets, defined as 2 consecutive ectopic beats; and 4, VT.

### In vivo cardiac function

Echocardiography was performed in conscious mice, using a single-handed manual restraint method, with an Acuson Sequoia C512 equipped with a 13 MHz (15L8) linear transducer.^15,25,26^ M-mode images in the parasternal short-axis view of the LV were used to evaluate LV chamber diameter and fractional shortening.^25,26^

LV hemodynamics and pressure-volume loops were obtained in anesthetized mice (isoflurane, 1.5%) in the closed chest preparation with an MPVS Ultra system for small animals (Millar Instruments), equipped with an SPR-839 catheter and coupled to a PowerLab 8/35 A/D converter (ADInstruments).^23,24,26^ The mouse was warmed with a heat lamp; the right carotid artery was exposed, and the pressure transducer was inserted and advanced into the LV cavity. Data were acquired with LabChart 8 software (ADInstruments). For calibration of an SPR-839 catheter and evaluation of LV blood volume, the manufacturer’s instructions were followed. Briefly, cuvette calibration protocol with fresh heparinized, warm blood and in vivo bolus infusion of hypertonic saline solution (15% sodium chloride [NaCl]) were performed to compute slope and intercept and to assess parallel conductance.^19^ Analysis was performed using LabChart software.

### Ex vivo properties of the mouse heart

With the animal under deep anesthesia (isoflurane) and after administration of heparin (~200 units, i.p.), bilateral thoracotomy was performed, the ascending aorta was cannulated with PE50 tubing connected to a 23G ¾ needle, and the heart was excised. Subsequently, hearts were perfused in a Langendorff apparatus (Radnoti). Perfusion was accomplished at a constant pressure of ~80 mm Hg with prewarmed Krebs–Henseleit buffer (MilliporeSigma) containing, in mmol/L, 118 NaCl, 4.7 K^+^ chloride (KCl), 11 glucose, 1.2 magnesium sulfate, 1.2 monopotassium phosphate, 1.8 calcium chloride (CaCl_2_), and 25 sodium bicarbonate, gassed with 95% oxygen and 5% carbon dioxide (pH 7.4) at 37°C. The temperature was maintained by immersing the heart in a water-heated glassware reservoir (Radnoti), containing preheated Krebs–Henseleit buffer.^27,28^

To assess the electrical activity of perfused hearts, epicardial monophasic APs (MAPs) were recorded using a micro MAP-tip electrode (Harvard Apparatus) positioned on the LV free wall.^19,28^ For infarcted hearts, the MAP-tip electrode was positioned on the surviving remote myocardium of the LV free wall, away from the scarred tissue. A 2-lead mini ECG system (Harvard Apparatus), in which electrodes were placed on the right atrium and apex of the heart, was used to obtain a pseudo-ECG.^15,19^ Electrical signals were amplified (6600 Amplifier, Gould Instruments), digitized using a 160 kHz A/D converter (DI-1120 HS, Dataq), and recorded with WinDaq software (Dataq).

Programmed electrical stimulation (PES) was introduced to assess the propensity of the mouse heart to develop ventricular arrhythmias.^5,19,29^ A stimulus generator (STG2004, Multi Channel Systems) controlled by a PC was coupled with a stimulus isolation unit (ISO-STIM 01M, npi) to apply PES protocols. Initially, a train of 20 pacing stimuli (S1) applied at 125 ms cycle length with an extra stimulus (S2) inserted at the end of the train was used. The S1–S2 interval was progressively reduced until the S2 stimulus either failed to generate an AP or induced ectopic events.^5,19^ In a subset of perfused hearts, a second protocol consisted of a 10-second stimulation train at constant frequency followed by a 10-second rest period. Trains of progressive higher frequency were applied till ectopic events occurred or the heart failed to follow the stimulation.^5,19^ The appearance of ventricular ectopic beats, VT (3 or more consecutive ectopic beats), and/or ventricular fibrillation (VF) was established.^19,29^ Data were analyzed with LabChart8 software.

### Histologic analysis and assessment of myocardial infarct size

Images of formalin-fixed transverse sections of the LV at the midventricular level were collected with a stereo microscope (SW-3T13X, AmScope) equipped with a digital camera (MU1003, AmScope). Infarct size was quantified using the midline length approach.^15,30^ Briefly, using ImageJ software, a line was manually drawn at the midpoint of the surviving myocardium between the epicardial and endocardial surfaces, and another line was drawn at the level of the thin transmural scar tissue. These lines formed a circumference. Infarct size was calculated as the ratio of the midline length at the scar tissue to the total midline circumference and expressed as a percentage.

For a subset of infarcted hearts, transverse LV sections at the midventricular level were embedded in paraffin and sliced into thin sections (~4 mm thickness).^15,21^ Fibrotic tissue was detected using Masson’s trichrome staining (Masson’s trichrome stain kit, MasterTech, StatLab), following the manufacturer’s instructions.^15,23^ Images were captured using a stereo microscope (SW-3T13X, AmScope) equipped with a digital color camera (MU1003, AmScope). Images were used to evaluate fibrotic area size using the area approach.^15,30^ Using ImageJ, the areas of fibrotic tissue and the total area of the LV section were manually traced. Fibrotic tissue size was expressed as the percentage of the total fibrotic area relative to the total LV area.

### Isolation of ventricular cardiomyocytes

With the animal under deep anesthesia (isoflurane) and after administration of heparin (~200 units, i.p.), bilateral thoracotomy was performed, the heart was excised, and LV myocytes were enzymatically dissociated as previously reported.^23,24^ Briefly, the heart was connected to a plastic cannula for retrograde perfusion through the aorta in a Langendorff system (Radnoti), at 37°C. Perfusate consisted of a Ca^2+^-free solution gassed with 100% oxygen. After 5 minutes, 0.1 mmol/L CaCl_2_, 274 units/mL collagenase (type 2, Worthington Biochemical Corp), and 0.57 units/mL protease (Type XIV, Sigma-Aldrich) were added to the solution, which contained, in mmol/L, NaCl 126, KCl 4.4, magnesium chloride (MgCl_2_) 5, HEPES 20, glucose 22, taurine 20, creatine 5, sodium pyruvate 5, and monosodium phosphate 5 (pH 7.4). At completion of digestion, atria and right ventricle were dissected and discarded. The remaining LV free wall and septum were separated. For infarcted hearts, the scarred tissue was removed from the LV free wall. Tissue was cut into small pieces, and these fragments were shaken in resuspension solution and filtered using a 230 μm sieve (Sigma-Aldrich). For physiological studies, myocytes from the surviving portion of the LV free wall of infarcted hearts and cells from corresponding regions of noninfarcted hearts were used. For physiological tests, only rod-shaped LV free wall myocytes exhibiting cross striations and showing no spontaneous contractions or contractures were selected; cells were used within 8 hours after enzymatic digestion.

### Whole-cell patch-clamp studies

Isolated adult LV myocytes were placed in a bath on the stage of a Zeiss Axiovert 200 microscope for patch-clamp measurements.^13,31^ Data were acquired using the whole-cell patch-clamp technique in voltage-clamp modes using Multiclamp 700B amplifiers (Molecular Devices). Electrical signals were digitized using a 250 kHz 16-bit resolution A/D converter (Digidata 1550B, Molecular Devices) and recorded using pCLAMP 10 software (Molecular Devices) with low-pass filtering at 2 kHz. Membrane capacitance (C_m_) was measured in voltage-clamp mode using a 5 mV voltage step and pCLAMP software algorithm; this parameter was used to normalize transmembrane currents.^5,13^ Pipettes were pulled using a horizontal glass microelectrode puller (P-97, Sutter Instruments); when filled with intracellular solution, pipettes had a resistance of 1–2 MΩ.

In adult cardiomyocytes, transient outward Kv currents were assessed with a previously reported protocol.^5,13,32^ Cells were bathed with a modified Tyrode solution containing (in mM) NaCl 140, KCl 4, MgCl_2_ 1, HEPES 10, glucose 10, CaCl_2_ 1.2, and 0.3 mM CdCl_2_ to block L-type Ca^2+^ current (pH 7.4, adjusted with sodium hydroxide).^5,33^ The composition of the pipette solution was (in mM) NaCl 5, KCl 20, K^+^ aspartate 120, MgCl_2_ 1, magnesium-adenosine triphosphate 5, EGTA 10, and HEPES 10 (pH 7.2 with K^+^ hydroxide).^5,33^ After membrane capacitance and series resistance compensation (≥70%), Kv currents were activated using depolarizing voltage steps from holding potential (V_h_) of −80 mV to +60 mV, 4.5 seconds in duration. The loss of voltage control was minimized owing to the proximity to the reversal potential of Na^+^. The decay phases of outward currents evoked by 4.5-second depolarization steps were analyzed with a triexponential function to evaluate the amplitude of components of *I*_to_ with fast (*I*_to,f_) and slow (*I*_to,s_) inactivation kinetics, and *I*_K,slow_, based on their distinct time constants of decay together with a sustained (noninactivating) component (*I*_ss_).^1,5,13,32,33^ For cells lacking *I*_to,f_ or *I*_to,s_, a biexponential function was used. Interpulse interval was 5.5 seconds. Currents were normalized by *C*_m_ and expressed as pA/pF.

### Western blotting

Whole protein extracts from snap-frozen LV cardiomyocytes or LV tissue were prepared using radioimmunoprecipitation assay lysis and extraction buffer (Thermo Fisher Scientific) supplemented with a protease and phosphatase inhibitor cocktail (Thermo Fisher Scientific), as previously reported.^19,23,34^ A total of 50 μg of protein was loaded and separated using either a 15% or 8% sodium dodecyl sulfate polyacrylamide gel electrophoresis. Proteins were then transferred to nitrocellulose membranes using a TransBlot Turbo system (Bio-Rad). Membranes were blocked with either 5% nonfat dry milk or 5% bovine serum albumin in TBS-Tween for 1 hour at room temperature. Membranes were then incubated overnight at 4°C with the primary antibodies. Membranes were then incubated with horseradish peroxidase–conjugated secondary antibodies (Cell Signaling Technology, catalog #7076S and 7074S). The blots were developed using an enhanced chemiluminescence detection system (Thermo Fisher Scientific, catalog # 34076). An iBright imaging system (Thermo Fisher Scientific) was used for signal detection. The optical density of the bands was measured using the ImageJ software and normalized against the expression levels of either glyceraldehyde-3-phosphate dehydrogenase or Vinculin as loading controls. The following primary antibodies were used: Notch1 (rabbit monoclonal, Abcam, ab52627), Kv channel–interacting protein 2 (KChIP2) (rabbit polyclonal, Thermo Fisher Scientific, PA5–41075), glyceraldehyde-3-phosphate dehydrogenase (mouse monoclonal, Thermo Fisher Scientific, AM4300), and Vinculin (rabbit monoclonal, horseradish peroxidase conjugated, Cell Signaling Technology, 18799S).

### Quantitative reverse transcription polymerase chain reaction

Total RNA was prepared from mouse LV isolated cardiomyocytes using the RNeasy Mini Kit (Qiagen) following the manufacturer’s instructions, as previously reported.^19,23^ Complementary DNA (cDNA) was obtained from total RNA using the Transcriptor High Fidelity cDNA Synthesis Kit (Sigma-Aldrich). Real-time reverse transcription polymerase chain reaction (PCR) was performed with primers listed in Supplemental Table 1. The QuantStudio real-time PCR system (Applied Biosystems) was used for quantitative reverse transcription PCR. In each case, cDNA was combined with Fast SYBR^™^ Green Master Mix (Applied Biosystems) in a 25 μL reaction mix. Cycling conditions were as follows: 95°C for 10 minutes followed by 40 cycles of amplification (95°C denaturation for 10–15 seconds, 60°C annealing and extension for 1 minute). The melting curve was then obtained. C_t_ values were normalized with respect to the housekeeping gene hypoxanthine guanine phosphoribosyl transferase. Data were expressed as relative quantity (2^−ΔCt^) with respect to the housekeeping gene.

### Statistical analysis

Data are presented in the text as fold or percentage change based on the ratio of median values. Graphically, data are presented as scattered plots with indication of median and interquartile ranges, unless otherwise specified. Statistical analysis was performed using GraphPad Prism 10. Data were initially tested for normality (Shapiro-Wilk) and equal variance to determine whether parametric or nonparametric analysis should be applied. Student’s *t* test, paired *t* test, or Mann-Whitney rank sum test was used for comparison between 2 groups.^5,23,35^ For comparison of data involving 2 factors (ie, genotype and MI), 2-way analysis of variance followed by uncorrected Fisher’s least significant difference test for multiple comparisons or by Tukey’s multiple comparisons test.^23^ For survival curves, Gehan–Breslow–Wilcoxon and log-rank (Mantel–Cox) tests were used.^19^ For categorical data analysis, Fisher’s exact or χ^2^ tests for trend were used.^19,23^ *P* < .05 was considered significant. Unless otherwise specified, asterisks are used in graphical representations to denote *P* values: *P < .05; **P < .01; ***P < .001; ****P < .0001.

## Results

### Loss of the *Notch1* gene minimally affects the functional defects of infarcted hearts

To establish the contribution of Notch1 receptor to the function of the adult heart in normal and diseased conditions, double transgenic mice with tamoxifen-inducible MerCreMer recombinase expression leading to *Notch1* gene deletion in cardiomyocytes (cKO) were obtained by intercrossing single transgenic lines.^16,18^ The inducible knockout model was implemented to circumvent developmental defects and embryonic lethality associated with the constitutive loss of the *Notch1* gene.^17^ Single transgenic mice homozygous for the MerCreMer construct were treated with tamoxifen and used as WT controls.^18,19^ Male and female mice treated with tamoxifen were studied in the naïve state and after myocardial ischemic injury, a condition associated with transient, de novo re-expression of Notch1 receptor in the heart.^9,10,12^

In WT and cKO male and female mice, MI, induced by permanent coronary artery ligation, resulted in the loss of contraction of the anterior wall of the LV, progressive dilation of the LV chamber, and reduction of LV fractional shortening (Figure 1). For both male and female animals, echocardiographic, anatomic, and functional parameters were comparable between WT and cKO mice in the naïve state and after MI.

To further evaluate the consequences of *Notch1* gene deletion on the functional defects of the heart after ischemic injury, LV hemodynamics and pressure-volume loops were obtained in mice in the naïve state and at ~4 weeks after MI. In WT and cKO male mice, MI reduced developed pressure, attenuated the maximal rate of pressure development (dP/dt_max_), and prolonged the time constant (tau) of pressure decay (Figure 2A and 2C). In addition, LV end-diastolic volume was increased after MI, together with a reduction in stroke volume, cardiac output, and ejection fraction (Figure 2B and 2D). Alterations occurring in male mice after MI were also detected in female animals (Figure 2E and 2F). Importantly, for both sexes, LV hemodynamic and volumetric parameters were comparable between WT and cKO mice in the naïve state and after MI.

To contextualize the functional properties of injured hearts from WT and cKO mice, infarct size was evaluated in LV sections obtained at the midventricular level. A subset of the sections was also processed histologically and stained to identify fibrotic tissue (Supplemental Figure 2A). Infarct size was approximately 37% in WT and 39% in cKO mice (Supplemental Figure 2B). In addition, the fibrotic tissue area represented ~36% and ~30% of the total LV section area in WT and cKO hearts, respectively (Supplemental Figure 2C).

Thus, ischemic injury induces LV dilation and deterioration of systolic and diastolic indices of cardiac function. The loss of the *Notch1* gene seems to have minimal or no influence on the anatomic and functional alterations occurring in the heart after MI.

### Loss of the *Notch1* gene attenuates the electrical remodeling of cardiomyocytes and myocardium of infarcted hearts

Notch signaling activation in cardiomyocytes reduces transient outward K^+^ currents and prolongs the duration of the AP,^12,13^ raising the possibility that re-expression of the Notch receptor after ischemic injury contributes to the electrical remodeling of cardiomyocytes and myocardium of the infarcted heart. To test this possibility, the effects of *Notch1* gene deletion on the AP profile of infarcted hearts from male and female mice were evaluated ex vivo, using isolated organ preparations.

In perfused hearts electrically stimulated at an 8 Hz pacing rate, the duration of the epicardial MAP, measured at 50% of repolarization, was comparable in the LV myocardium of hearts from WT and cKO naïve male mice. At ~4 weeks after MI, compared with intact hearts, the MAP duration recorded in the remote myocardium of the LV free wall, away from the scarred tissue, was prolonged by 36% and 19% in hearts from WT and cKO male mice, respectively. Thus, after MI, the duration of the MAP was shorter in hearts from cKO mice, with respect to WT animals (Figure 3A and 3B). Similar results were observed in hearts from female mice (Figure 3C).

To assess the role of repolarizing K^+^ currents in the reduced AP remodeling observed in infarcted hearts of *Notch1*-deficient mice, transient outward Kv currents were assessed in LV myocytes obtained from WT and cKO female mice, both in the naïve state and 14–17 days after MI. Membrane capacitance, which was used to normalize current densities, was comparable in myocytes from WT and cKO naïve mice, and it increased by ~42% in cells from infarcted hearts for both genotypes (Supplemental Figure 3). Kv current components were computed by analyzing the decay phase of the total outward current evoked by a depolarization step in voltage-clamp mode. In particular, a triexponential function was used to dissect *I*_to,f_, *I*_to,s_, and *I*_K,slow_, based on their distinct time constants of decay, which differ by an order of magnitude, together with the evaluation of the steady (noninactivating) component (*I*_ss_).^5,13,32^ Total and individual transient outward Kv current densities did not differ in myocytes from WT and cKO naïve mice. However, in myocytes from infarcted hearts, individual Kv current components and*I*_ss_ were reduced by 21%–66% in myocytes from WT mice. This resulted in a 47% reduction in total Kv current density in WT myocytes after MI (Figure 4A and 4B). In contrast, no significant alterations of Kv currents occurred after MI in myocytes from cKO mice. Therefore, with MI, total Kv current density was larger in myocytes from cKO mice, with respect to cells from WT animals.

Given that Notch signaling functions as a transcriptional repressor of target genes, including those encoding Kv channel subunits,^12,14^ molecular analyses were performed to determine whether MI, either alone or in combination with the loss of the *Notch1* gene, affects the expression of Kv channels. In the surviving LV myocardium of infarcted WT hearts, transcript levels of *Kcnd2*, *Kcna5*, and *Kcnip2* genes, which encode, respectively, K channel subunits Kv4.2 and Kv1.5 and KChIP2, were reduced by 69%, 54%, and 50% compared with LV tissue from naïve WT mice. Interestingly, for cKO animals, expression of *Kcnd2* and *Kcna5* remained unchanged in the surviving LV myocardium of infarcted hearts, with respect to the LV tissue from naïve hearts, whereas the expression of *Kcnip2* was reduced by 35% (Figure 5A). Similar trends for KChIP2 expression were found by Western blotting (Figure 5B and 5C).

Thus, the loss of *Notch1* in cardiomyocytes prevents the reduction of repolarizing Kv currents and attenuates the prolongation of the AP profile occurring after MI.

### Loss of the *Notch1* gene attenuates the occurrence of electrical disturbances of infarcted hearts

The prolongation of the AP after MI has important consequences for the heart’s vulnerability to arrhythmias.^6,36–38^ Thus, to establish whether silencing of Notch1 and the associated attenuation of electrical remodeling confer cardioprotective effects on postinfarction electrical stability, the impact of *Notch1* gene deletion on arrhythmia incidence and susceptibility was evaluated both in vivo and ex vivo.

ECG recordings in conscious mice revealed that arrhythmic events were rare in naïve WT and cKO mice of both sexes. However, at 1 day after MI, 69% of WT mice exhibited ectopic events, whereas only 31% of cKO animals presented similar disturbances (Figure 6A and 6B and Supplemental Figure 4A). The incidence of ectopic events was comparable between male and female animals for the 2 genotypes (Supplemental Figure 4B). Moreover, cKO mice presented a significantly lower cumulative arrhythmia score at 1 day after MI than their WT counterparts (Figure 6C). After the acute phase of the ischemic event, the occurrence of arrhythmias declined, and no differences were observed between WT and cKO mice. No differences in survival rate were observed between the 2 groups of mice after MI.

Alterations of the repolarization phase of the AP after MI favor the occurrence of reentrant arrhythmias.^36,37^ To investigate whether the attenuated electrical remodeling associated with *Notch1* gene deletion affects the susceptibility of the infarcted heart to develop reentrant circuits, isolated hearts were subjected to PES. In particular, intact and infarcted hearts were electrically activated by a stimulation protocol involving progressively shorter coupling intervals (S1–S2). At ~4 weeks after MI, infarcted hearts from WT mice exhibited a higher propensity to develop VT or VF than intact hearts from naïve WT mice. In contrast, infarcted hearts from cKO mice did not show a statistical increase in VT/VF susceptibility (Figure 6D and 6E).

To establish whether reduced ectopic events in cKO mice acutely after ischemic damage were coupled with preservation of electrical repolarization of the myocardium, a subset of hearts from WT and cKO mice at 1 day after MI were studied ex vivo. Notably, epicardial MAP duration, measured at 90% repolarization in the remote myocardium, was shorter in perfused hearts from cKO animals than in WT (Supplemental Figure 5A). Moreover, by incremental pacing protocols, cKO hearts had a reduced propensity to develop arrhythmia compared with WT hearts (Supplemental Figure 5B).

Thus, the loss of the *Notch1* gene mitigates the occurrence and propensity of arrhythmias after MI, which is accompanied by improved electrical stability of the postinfarcted heart.

## Discussion

Results of the current study demonstrate that deletion of the *Notch1* gene mitigates electrophysiological remodeling of the heart after MI, in parallel with a reduced incidence and susceptibility to arrhythmias.

Deletion of the *Notch1* gene did not affect cardiac function or electrophysiological properties in naïve animals, aligning with previous findings that Notch1 signaling is silenced in myocytes of the healthy, adult heart.^9–11,39^ Moreover, the loss of *Notch1* did not significantly alter the progression of LV chamber dilation, systolic and diastolic dysfunction, or reduced cardiac output after MI. Despite producing only minimal structural and functional alterations, *Notch1* deletion in infarcted mice prevented the reduction of Kv currents in myocytes measured at 2 weeks after MI and attenuated the prolongation of the LV myocardial AP observed both acutely and chronically. These electrophysiological alterations likely represent the mechanistic basis for the reduced incidence and susceptibility to post-MI arrhythmias observed in *Notch1*-deficient hearts. In fact, although necrotic regions and replacement fibrosis after MI are key structural substrates for the formation of reentrant circuits that sustain VT, the electrical remodeling of surviving myocytes plays a crucial role in promoting rhythm disorders.^40,41^ In fact, the prolongation of the AP duration (1) alters the refractory properties of the cardiac tissue, disrupting impulse propagation and facilitating reentry mechanisms^36,37^; (2) increases the beat-to-beat variability of the repolarization,^2^ thereby enhancing the susceptibility to rhythm disturbances^42^; (3) promotes early afterdepolarizations, which can trigger arrhythmias^36^; and (4) augments Ca^2+^ influx and intracellular Ca^2+^ loading, favoring spontaneous Ca^2+^ releases, delayed afterdepolarizations, and ectopic discharge.^38^ Thus, preservation of Kv current densities in myocytes and maintenance of AP properties in the myocardium are viewed as key contributors to the improved electrical stability of the infarcted heart in mice lacking the *Notch1* gene.

Transient outward Kv currents are the primary determinants of AP repolarization in rodent myocytes. In contrast, in cardiomyocytes from large mammals, these currents contribute mainly to the early phase of repolarization that precedes the plateau phase, thereby shaping the characteristic spike-and-dome morphology of the cardiac AP.^1,43^ These electrophysiological differences highlight the need for caution when extrapolating the impact of altered Kv currents on AP duration and arrhythmogenic potential from rodents to large mammals and humans.

Kv currents comprise components with rapid activation and fast (I_to,f_), intermediate (I_to,s_), and slow (I_K,slow_) kinetics of inactivation.^1,33^ The presence of these ionic currents in cardiomyocytes depends on the heteromeric assembly of Kv channel subunits, including Kv4.2, Kv4.3, Kv1.4, Kv1.5, Kv2.1, and KChIP2.^1,33^ Importantly, the heterogeneous distribution of Kv channel subunits in the myocardium of small and large mammals underlies the transmural gradient of the transient outward current in the LV and the regional difference of AP characteristics.^43,44^ Thus, preservation of Kv currents after MI likely prevents the disruption of the transmural repolarization gradient, thereby contributing to enhanced electrical stability.^7^

As reported in the current study, reduction of Kv currents and altered expression of Kv channel subunits in myocytes have been observed after MI in mice,^45^ rats,^46^ and large mammals.^47^ Importantly, under physiological and pathologic conditions, the prolongation of the AP associated with reduced Kv currents has positive inotropic effects,^2,5,27^ representing an adaptive cellular response aimed at preserving contractile function. However, as previously discussed, these electrophysiological alterations can predispose the myocardium to adverse electrical events and initiate maladaptive processes that contribute to heart failure progression.

The loss of viable, contracting myocardium after the ischemic insult leads to an inflammatory response and increases the loading condition of the LV.^48,49^ These factors activate a series of biochemical and mechanical stimuli promoting scar formation, progressive chamber dilation, expression of fetal genes, and myocardial hypertrophy.^50^ Although the signaling pathways involved in the downregulation of Kv currents in myocytes after infarction remain incompletely understood, the reactivation of fetal genes after MI suggests a potential role of Notch1 signaling in modulating Kv currents.

The area bordering the infarct represents a spatially confined region of the myocardium in which myocytes undergo robust transcriptional changes and marked electrical remodeling.^51–53^ These local alterations contribute to heterogeneity in electrical repolarization within the injured heart, thereby increasing its susceptibility to arrhythmias. Although the present study did not achieve the spatial resolution necessary to distinguish regional differences in AP properties or ion channel expression between the remote and border-zone myocardium, it remains possible that differential expression of Notch1 contributes to postinfarction electrical heterogeneity. This possibility warrants further investigation.

Notch signaling is a highly conserved pathway involved in cell fate specification and developmental processes in various organs, a function that is maintained, in mammals, by the interaction between the 4 Notch receptors (Notch 1–4) and 5 canonical ligands (Jag1, Jag2, and delta-like 1, 3, and 4).^54^ The Notch receptor-ligand interactions activate transcriptional responses that, in the heart, mediate cardiac patterning, cardiomyocyte differentiation, ventricular trabeculation, and valve and outflow tract development.^54^ Notch1 is expressed in the mouse heart starting at E8.5 and is found in ventricular myocytes at E12.5.^17^ Its expression persists in cardiomyocytes in the early neonatal stage, before being silenced in the adult myocardium.^13^ Interestingly, genetic approaches have revealed that activation of Notch1 signaling in adult cardiomyocytes represses Kv currents and prolongs the AP duration.^12,13^ Conversely, inhibition or silencing of Notch signaling in developing myocytes increases Kv currents,^13,14^ reinforcing a causal relationship between Notch1 signaling activation and Kv current downregulation, a mechanism that seems to be functional in the postinfarcted heart.

The signal transduction mechanisms linking Notch1 activation and the transcriptional repression of Kv channels in the context of MI remain to be elucidated. Notably, levels of the Notch ligand Jag1 are increased in the myocardium after ischemia,^9,10^ potentially triggering the canonical Notch1 signaling pathway. This involves proteolytic cleavage of the Notch1 intracellular domain by the γ-secretase complex, nuclear translocation of Notch1 intracellular domain, and its interaction with transcriptional regulators, including RBP-J.^54^ Multiple regulatory mechanisms likely contribute to the transcriptional repression of Kv channel subunits. Previous studies have shown that activation of Notch1 signaling suppresses the expression of *Kcnip2*, *Kcnb1*, and *Kcna5* through an RBP-J–dependent mechanism by reducing trimethylation of histone H3 at lysine 4, an epigenetic mark associated with active transcription, at the proximal promoters of these genes.^12^ However, whether the loss of Notch1 signaling regulates Kv channel expression after MI through trimethylation of histone H3 at lysine 4–dependent mechanisms remains to be established.

We found that components of the transient outward Kv currents with fast, slow, and intermediate kinetics of inactivation were reduced in myocytes from infarcted WT mice, but not in cells from mice lacking the *Notch1* gene. These findings suggest that the preservation of Kv currents contributes to the maintenance of the AP duration in *Notch1*-null mice after MI. Moreover, molecular analyses revealed that *Notch1* deletion prevented the post-MI reduction of *Kcnd2* and *Kcna5* transcripts, which encode for Kv channel subunits Kv4.2 and Kv1.5, respectively, thereby suggesting the putative mechanistic basis for the preserved Kv currents in myocytes from *Notch1*-deficient infarcted mice. Interestingly, *Kcnip2* mRNA levels were similarly reduced in the myocardium of WT and *Notch1*-null mice after MI, and protein levels followed similar trends. These findings suggest that additional signaling mechanisms may regulate Kv current modulation independently of Notch1. Thus, more extensive analysis seems to be necessary to define the relative contribution of Notch1 signaling to Kv channel remodeling after MI. In this regard, signaling pathways involving α- and β-adrenergic receptors, calcineurin, nuclear factor of activated T cells 3, and nuclear factor-κB, which are activated after ischemia and function as transcriptional modulators of Kv channel–related genes,^45,55^ may have contributed to the post-MI Kv channel expression profile in WT and *Notch1*-null mice. Moreover, although not directly examined in this study, we cannot definitively rule out the possibility that upregulation of Notch2-4 receptors in the myocardium after ischemic injury,^10^ along with functional redundancy among Notch receptors,^56^ may have mitigated some of the effects associated with *Notch1* deficiency.

In the current study, the density of repolarizing Kv currents in myocytes was evaluated at 2 weeks after MI, whereas transcripts for Kv channel subunits and repolarization properties were assessed in the myocardium at 4 weeks after MI. Although the changes observed in ionic-current and AP properties in WT and *Notch1*-deficient mice likely arise from the same remodeling mechanisms, the temporal mismatch between these evaluations represents a limitation of the present investigation. In particular, the possibility that factors other than the Kv current may have contributed during the progression of the post-MI electrical remodeling cannot be excluded.

Despite the advances in reperfusion therapy and myocardial salvage, arrhythmias remain a frequent complication after acute MI. Approximately 6% of patients experience VT or VF within 48 hours of hospital admission.^57^ Moreover, among MI survivors with LV dysfunction or heart failure, the risk of sudden cardiac death within the first 30 days after infarction is 1.4%.^58^ Although structural damage and scar formation offer limited therapeutic options, interventions targeting myocyte electrical remodeling may represent a promising approach to reduce post-MI arrhythmia. Findings from the present study support the potential of Notch signaling as a therapeutic target in patients with ischemic heart disease.

## Supplementary Material

1

## Figures and Tables

**Figure 1:**
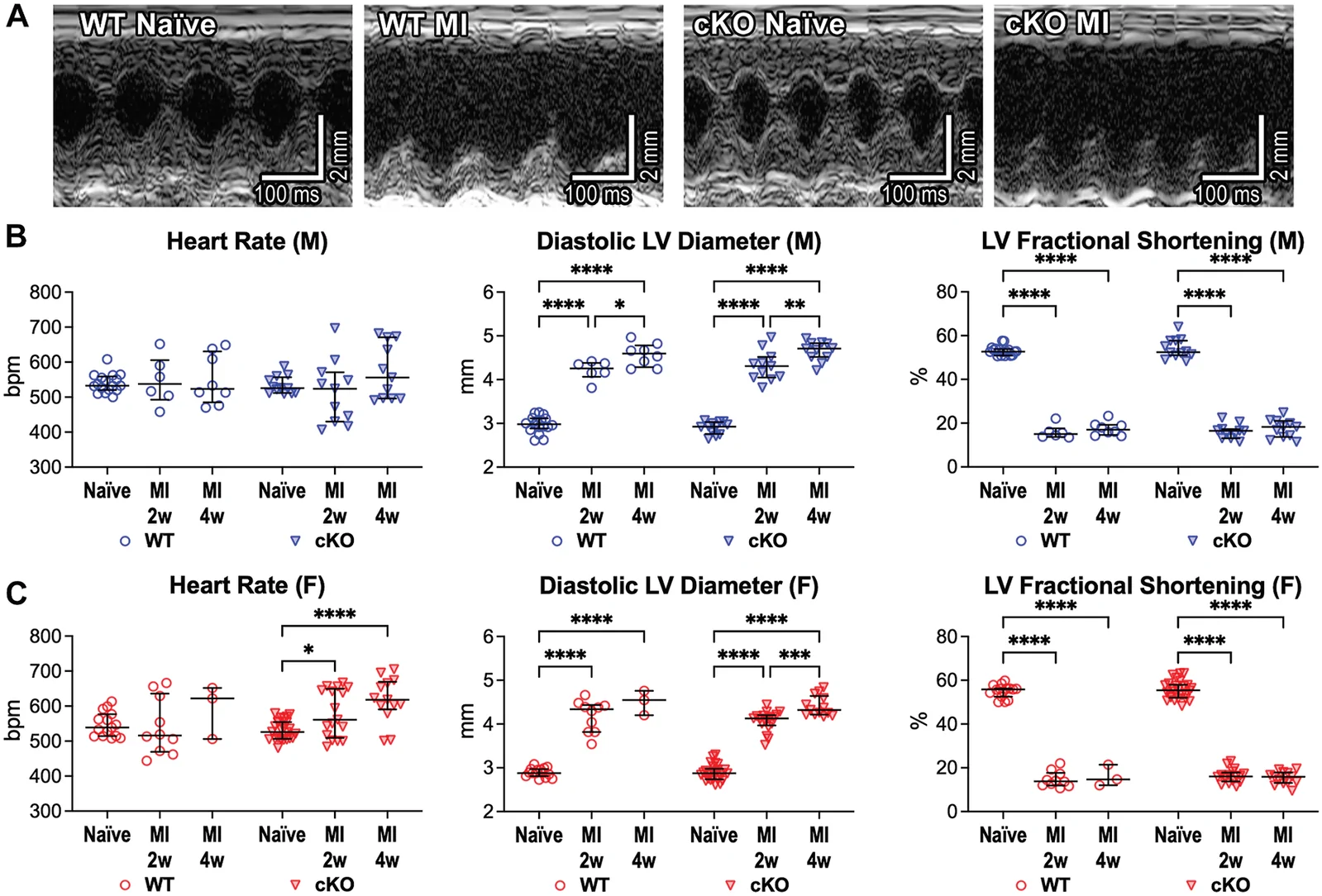
Loss of the *Notch1* gene does not affect the anatomy and function of the normal and infarcted hearts. **A:** M-mode echocardiographic images obtained in conscious WT and cKO male mice in the naïve state and at 4 weeks after MI. **B:** Data for LV anatomic and functional parameters obtained by echocardiography in male mice in the naïve state (3–4 months of age) and at 2 weeks (2w) and 4 weeks (4w) after MI. Data were obtained in male WT (naïve n = 17; 2w n = 6; and 4w n = 8) and male cKO mice (naïve n = 12; 2w n = 11; and 4w n = 11). Data are median and interquartile ranges with dot plots. *P < .05, **P < .01, ***P < .001, ****P < .0001 using 2-way ANOVA followed by Tukey’s multiple comparisons test. **C:** Quantitative data for echocardiographic parameters in female WT (naïve n = 15; 2w n = 10; and 4w n = 3) and female cKO mice (naïve n = 26; 2w n = 17; and 4w n = 13). Data are median and interquartile ranges with dot plots. *P < .05, ***P < .001, ****P < .0001 using 2-way ANOVA followed by Tukey’s multiple comparisons test. ANOVA = analysis of variance; cKO = conditional knockout; F = female; LV = left ventricle; M = male; MI = myocardial infarction; WT = wild-type.

**Figure 2:**
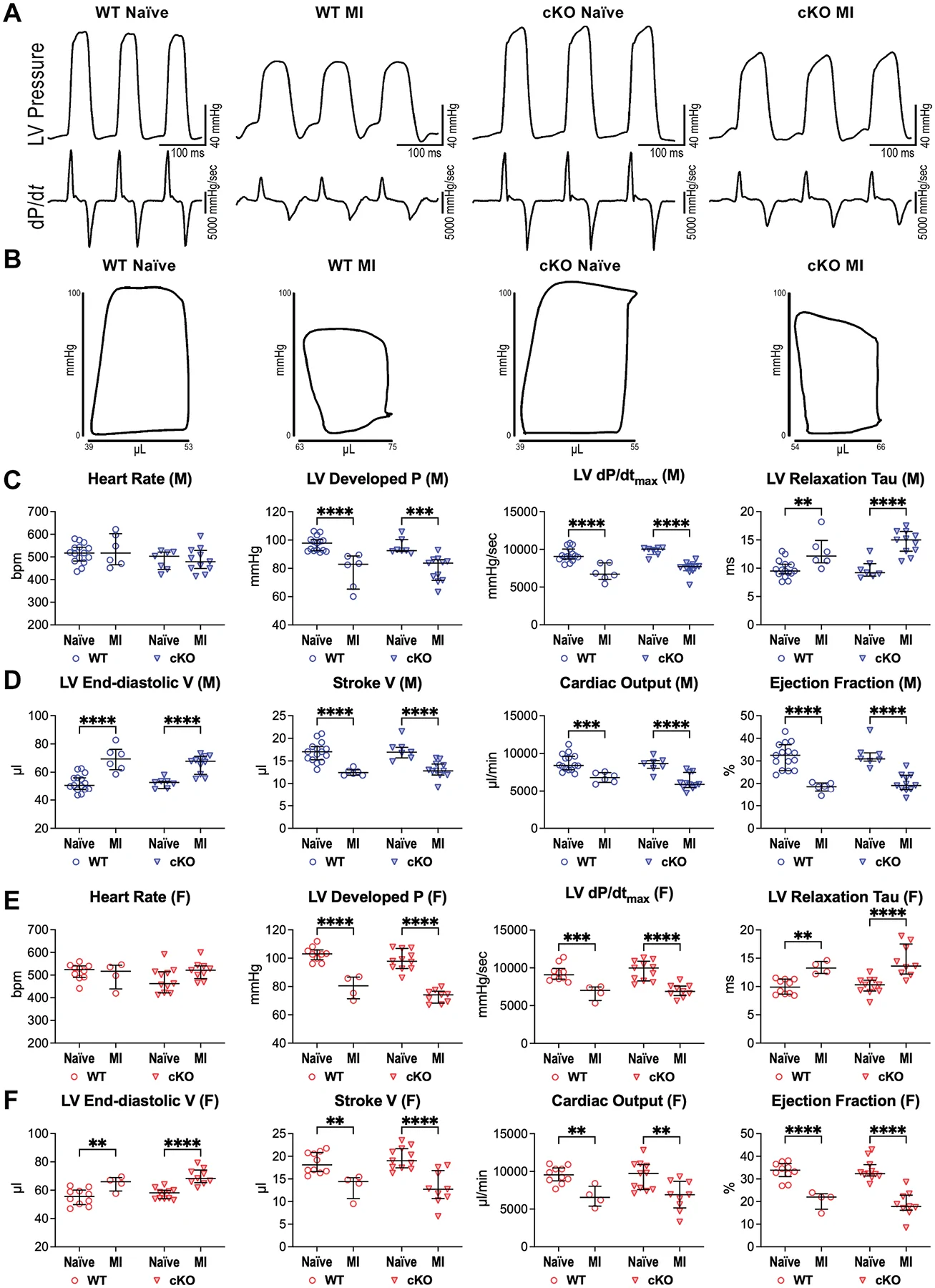
Loss of the *Notch1* gene does not affect LV hemodynamics and PV loops of the normal and infarcted hearts. **A and B:** Traces of LV pressure and its first derivative (dP/dt) (**A**) and PV loops (**B**) obtained in WT and cKO male mice in the naïve state and at 4 weeks after MI. **C and D**: Data for LV hemodynamic and volumetric parameters assessed by PV catheterization in male mice in the naïve state and at 4 weeks after MI. Data were obtained in WT (naïve n = 15; MI n = 6) and cKO male mice (naïve n = 7; MI n = 11). Data are median and interquartile ranges with dot plots. **P < .01, ***P < .001, ****P < .0001 using 2-way ANOVA followed by uncorrected Fisher’s LSD test for multiple comparisons. **E and F**: Quantitative data for LV hemodynamic and volumetric parameters in female mice in the naïve state and at 2–4 weeks after MI. Data were obtained in WT (naïve n = 10; MI n = 4) and cKO female mice (naïve n = 11; MI n = 9). Data are median and interquartile ranges with dot plots. **P < .01, ***P < .001, ****P < .0001 using 2-way ANOVA followed by uncorrected Fisher’s LSD test for multiple comparisons. ANOVA = analysis of variance; cKO = conditional knockout; LSD = least significant difference; LV = left ventricle; MI = myocardial infarction; PV = pressure-volume = V = volume; WT = wild-type.

**Figure 3:**
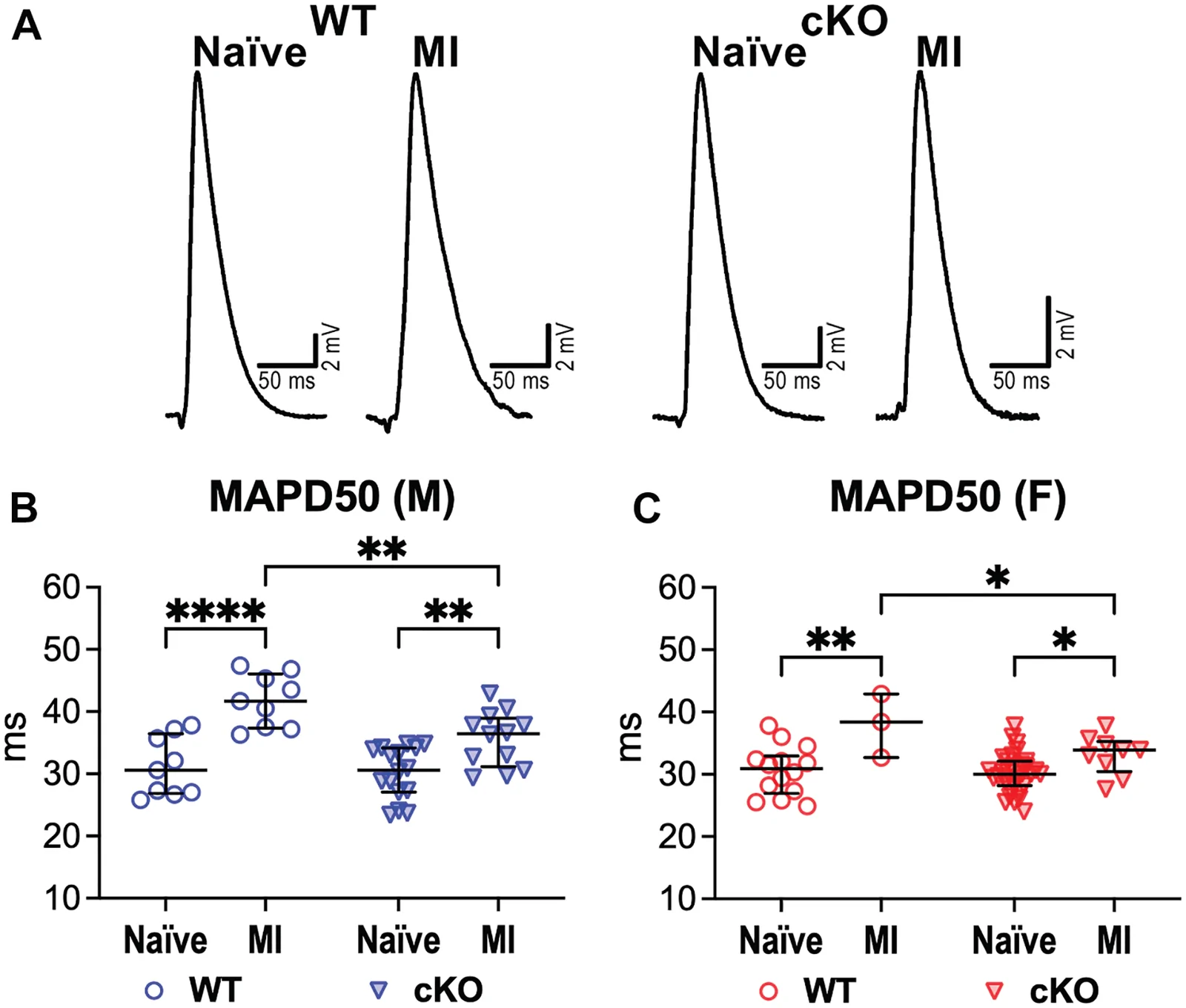
Loss of the *Notch1* gene attenuates the electrical remodeling of the LV myocardium after MI. **A:** Epicardial monophasic APs collected in the LV free wall of perfused hearts stimulated at 8 Hz. Traces are from WT and cKO male mice in the naïve state and at 4 weeks after MI. In infarcted hearts, monophasic APs were obtained in the remote LV myocardium. **B:** Data for the duration of the MAP50 in the LV myocardium of WT (naïve n = 9; MI n = 9) and cKO male mice (naïve n = 16; MI n = 12). MI refers to hearts at 4 weeks after MI. Data are median and interquartile ranges with dot plots. **P < .01, ****P < .0001 using 2-way ANOVA followed by uncorrected Fisher’s LSD test for multiple comparisons. **C:** Quantitative data for the duration of the MAP50 obtained in the LV myocardium of WT (naïve n = 14; MI n = 3) and cKO female mice (naïve n = 31; MI n = 9). Infarcted hearts were studied at 4 weeks after permanent coronary artery ligation. Data are median and interquartile ranges with dot plots. *P < .05, **P < .01 using 2-way ANOVA followed by uncorrected Fisher’s LSD test for multiple comparisons. ANOVA = analysis of variance; AP = action potential; cKO = conditional knockout; LSD = least significant difference; LV = left ventricle; MAP50 = monophasic action potential at 50% repolarization; MI = myocardial infarction; WT = wild-type.

**Figure 4:**
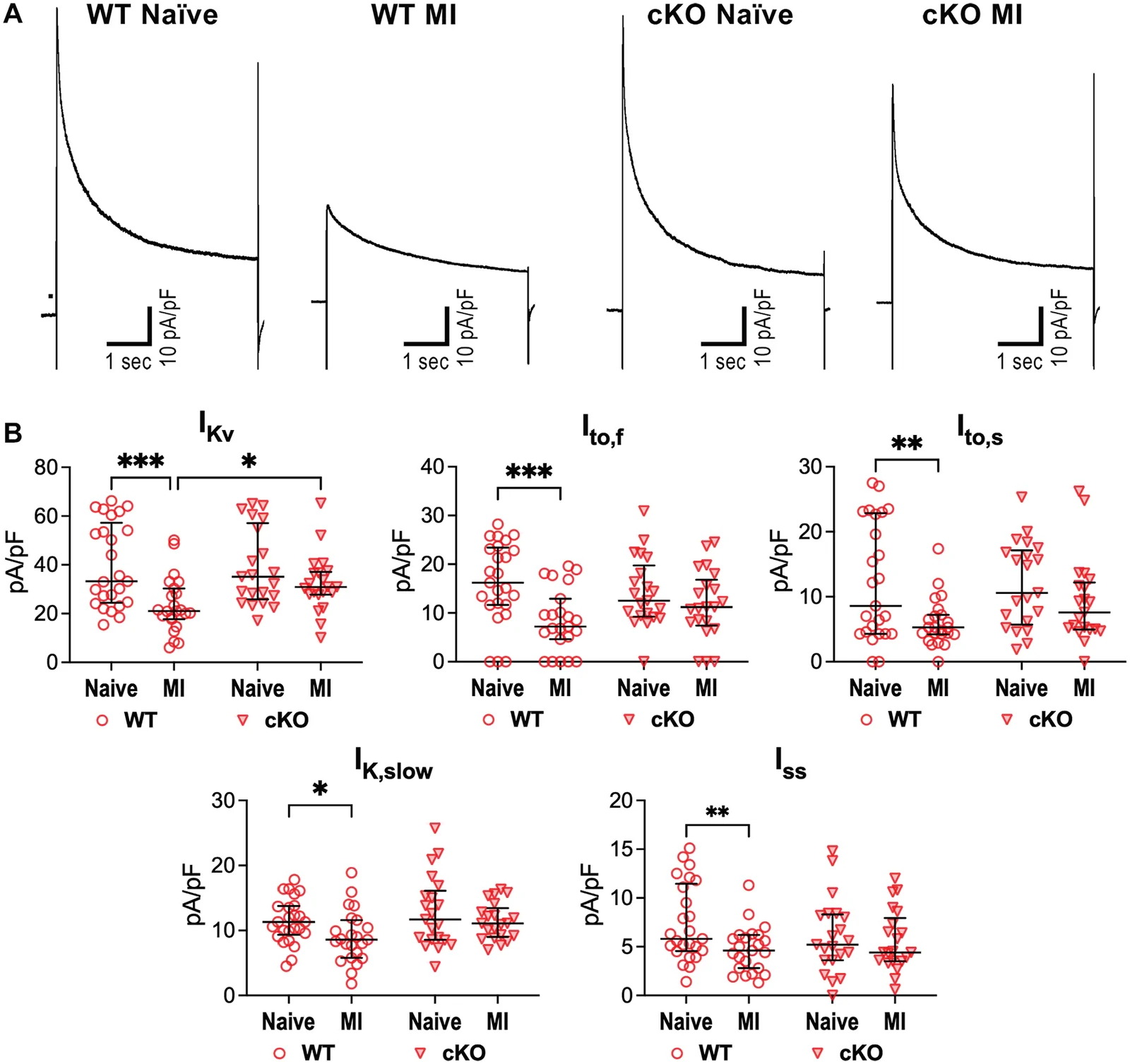
Loss of the *Notch1* gene attenuates the reduction of repolarizing Kv current in myocytes of the postinfarcted heart. **A:** Whole-cell voltage-gated K^+^ currents in LV myocytes from WT and cKO mice in the naïve state and 2 weeks after MI. **B:** Data for amplitude of Kv currents and individual components *I*_to,f_, *I*_to,s_, *I*_K,slow_, and *I*_ss_ for LV myocytes from WT (naïve n = 25 cells from 4 mice; MI n = 23 cells from 4 mice) and cKO female mice (naïve n = 21 cells from 4 mice; MI n = 21 cells from 4 mice). MI refers to cells from hearts at 2 weeks after MI. Data are median and interquartile ranges with dot plots. *P < .05, **P < .01, ***P < .001 using 2-way ANOVA followed by uncorrected Fisher’s LSD test for multiple comparisons. ANOVA = analysis of variance; cKO = conditional knockout; K^+^ = potassium; Kv = voltage-gated potassium; LSD = least significant difference; LV = left ventricle; MI = myocardial infarction; WT = wild-type.

**Figure 5:**
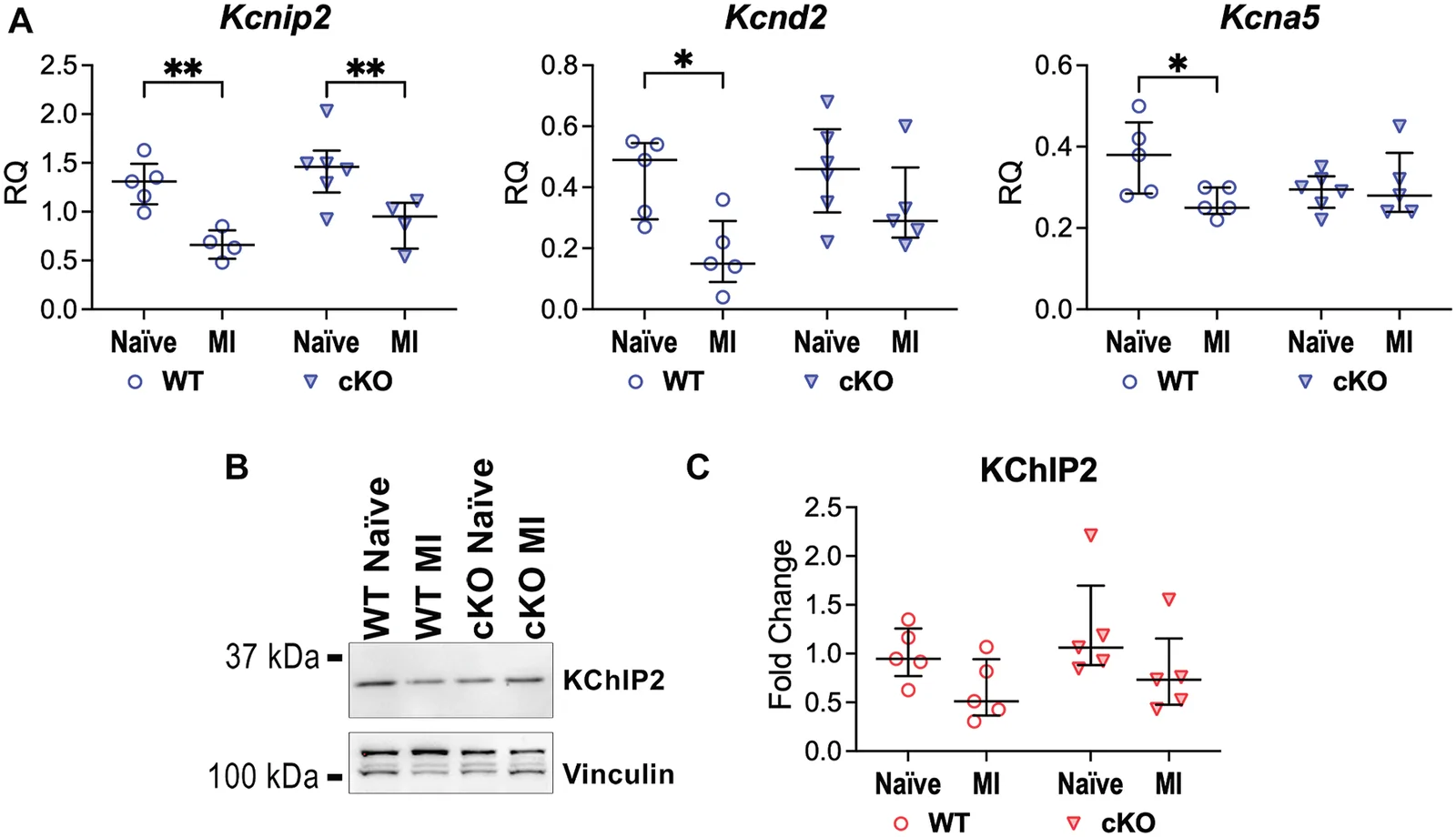
Loss of the Notch1 gene and expression of Kv channels after MI. **A**: Data for expression of genes related to channels mediating *I*_to_ (*Kcnip2* and *Kcnd2*) and *I*_K,slow_ (*Kcna5*) in the LV myocardium from WT (naïve n = 5; MI n = 4–5) and cKO male mice (naïve n = 6; MI n = 4–5). MI refers to hearts at 4 weeks after MI. Data are median and interquartile ranges with dot plots. *P < .05, **P < .01 using 2-way ANOVA followed by uncorrected Fisher’s LSD test for multiple comparisons. **B:** Expression of KChIP2 in LV myocytes from naïve female mice and surviving LV myocytes from the remote LV of mice at 2 weeks after MI. Vinculin is the loading condition. **C:** Quantitative data for the expression of KChIP2 in LV myocytes from WT (naïve n = 5; MI n = 5) and cKO female mice (naïve n = 5; MI n = 5). MI refers to myocytes from hearts at 2 weeks after MI. Data are median and interquartile ranges with dot plots. ANOVA = analysis of variance; cKO = conditional knockout; KChIP2 = Kv channel–interacting protein 2; Kv = voltage-gated potassium; LSD = least significant difference; LV = left ventricle; MI = myocardial infarction; WT = wild-type.

**Figure 6:**
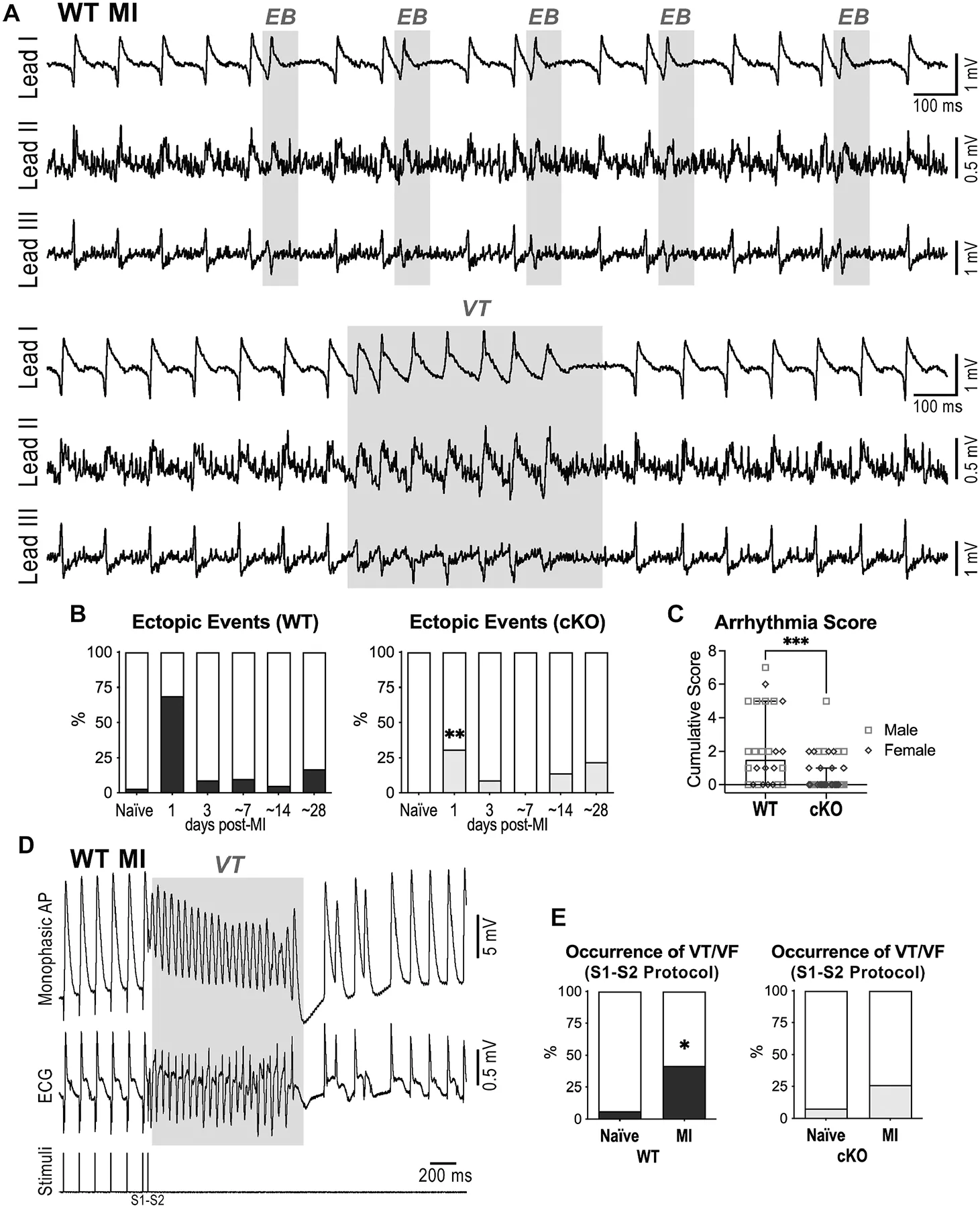
Loss of the *Notch1* gene reduces the occurrence of electrical disturbances after MI. **A:** ECG traces (leads I, II, and III) collected in a conscious WT male mouse at 1 day after MI. *Gray areas* identify EBs and VT. **B:** Quantitative data for the fraction of mice presenting ectopic events (solid segment) in the naïve state and at 1, 3, ~7, ~14, and ~28 days after MI for WT (n = 34, 26, 22, 21, 19, 12, respectively) and cKO male and female mice (n = 42, 42, 32, 35, 35, 27, respectively). Data are expressed as percentages of animals presenting ectopic events. **P < .01 vs WT at the same time point using Fisher’s exact test. **C:** Quantitative data for the combined arrhythmia score for WT (n = 26) and cKO male and female mice (n = 42) at 1 day after MI. Data are median and interquartile ranges with dot plots. *Gray square* and *black diamond symbols* identify data for male and female mice, respectively. ***P < .001 using the Mann-Whitney test. **D:** Traces reporting representative MAPs, pseudo-ECG (ECG), and stimulation protocol (Stimuli) for a heart from a WT male mouse at 30 days after MI. The extra stimulus originated from VT at a short S1–S2 interval. **E:** Quantitative data for the fraction of hearts presenting ectopic events (solid segment) by programmed electrical stimulation (S1–S2 protocol). Hearts obtained from naïve and infarcted WT (n = 32, 12, respectively) and cKO male and female mice (n = 38, 23, respectively). Data are expressed as a percentage of hearts presenting ectopic events. *P < .05 vs naïve using Fisher’s exact test. cKO = conditional knockout; EB = ectopic beat; ECG = electrocardiogram; MAP = monophasic action potential; MI = myocardial infarction; VF = ventricular fibrillation; VT = ventricular tachycardia; WT = wild-type.

## Appendix

### Supplementary Data

Supplementary data associated with this article can be found in the online version at https://doi.org/10.1016/j.hrthm.2026.03.1957.

## Abbreviations

AP: action potential
cKO: conditional knockout
KChIP2: Kv channel–interacting protein 2
LV: left ventricle
MAP: monophasic action potential
MI: myocardial infarction
PES: programmed electrical stimulation
VF: ventricular fibrillation
VT: ventricular tachycardia
WT: wild-type
