# Supplementary material for: Notch1 deletion reduces arrhythmogenic remodeling after myocardial infarction
Source: Heart Rhythm. Author manuscript; Available in PMC 2026 Nov 14. (PMC13769108; doi:10.1016/j.hrthm.2026.03.1957)
Supplement: Supplementary file 1 [file NIHMS2221932-supplement-1.pdf]

SUPPLEMENTAL MATERIAL

Notch1 Deletion Reduces Arrhythmogenic Remodeling after Myocardial Infarction

Emanuele Pizzo, PhD,[1] Daniel O. Cervantes, MD,[1] Giulia Piccinini, PhD,[1] Belinda Nyantakyi, MS,[1] Maria T. Ochoa, BSc,[1] Michael Y. Sun, BSc,[1] Andrea Filardo, PhD,[1] Harshada Ketkar, PhD,[2] Sudhir Jain, PhD,[2] Jason T. Jacobson, MD,[1,3] Malik Bisserier, PhD,[1] and Marcello Rota, PhD[1]

[1]Department of Cell and Molecular Physiology, New York Medical College, Valhalla, NY, USA; [2] Department of Pathology, Microbiology and Immunology, New York Medical College, Valhalla, NY, USA; [3]Department of Medicine, Westchester Medical Center, Valhalla, NY, USA.

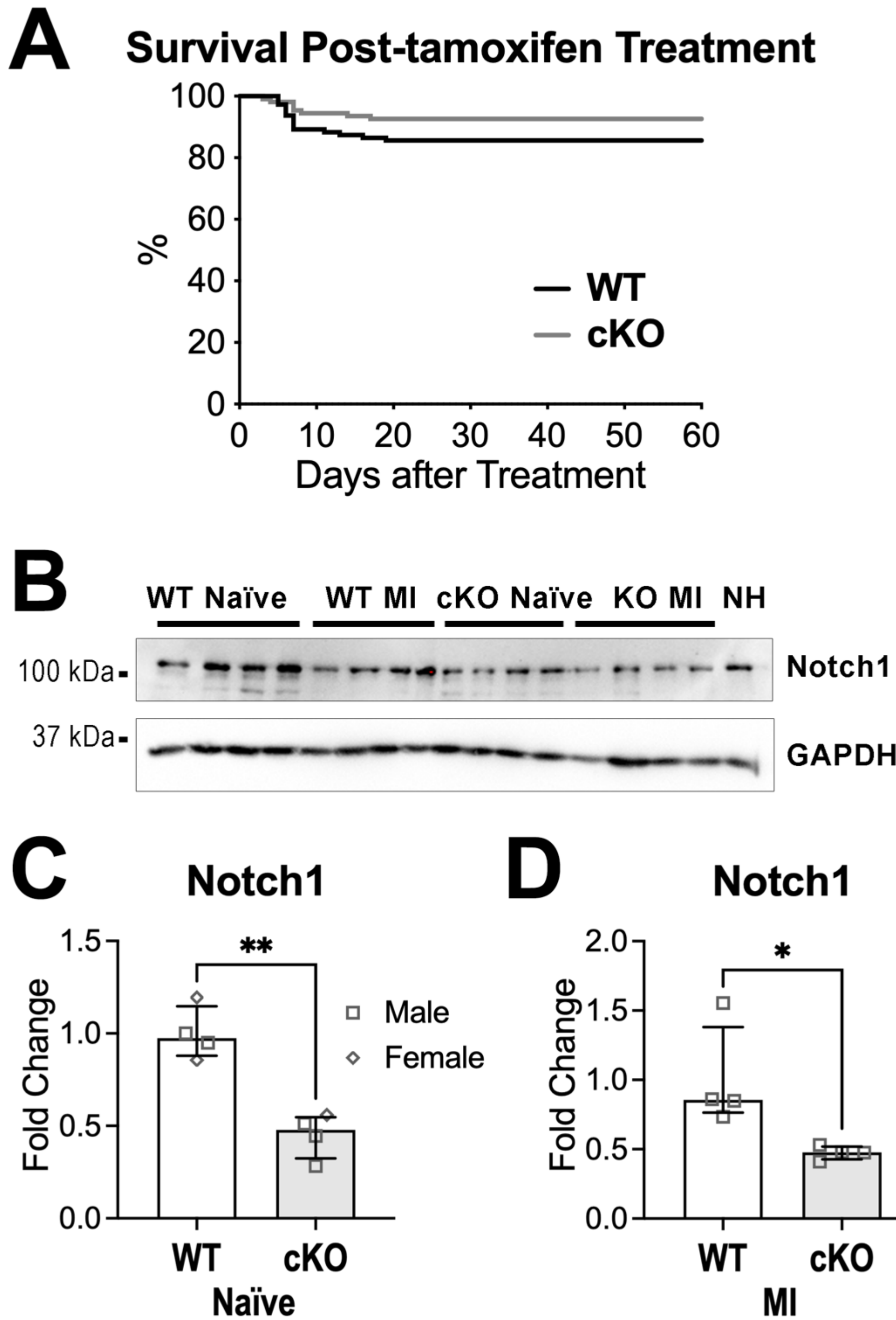

Supplemental Fig S1. Induction of gene recombination in mice. A, Survival curves following tamoxifen administration in male and female mice with cardiac-restricted, inducible expression of Cre recombinase alone or in combination with a floxed allele to achieve *Notch1* gene deletion. Single-transgenic homozygous mice expressing tamoxifen-inducible Cre recombinase under the α-myosin heavy chain promoter (wild-type, WT, n = 111; 41 males and 70 females) and double-transgenic mice homozygous for *Notch1* floxed gene (*Ntoch1$^{flox/flox}$*) and homozygous for MerCreMer construct (cKO, n = 108; 33 males and 75 females) are shown. Tamoxifen was administered via four injections on different days over a period of 4-8 days (30 mg/Kg of body weight/injection, i.p.). Survival curves were not significantly different between genotypes using Gehan–Breslow–Wilcoxon and log-rank (Mantel–Cox) tests. **B**, Expression of Notch1 in the LV myocardium of naïve and infarcted mice. GAPDH is the loading condition. NH, myocardium from neonatal heart. **C**, Expression of Notch1 proteins by Western blot in the myocardium (intraventricular septum) of naïve WT (n=4; 2 males and 2 female) and cKO (n=4: 3 males and 1 female) mice. Data are expressed as fold changes relative to WT and presented as median and interquartile ranges with dot plots. Square and diamond symbols identify data for male and female mice, respectively. **P<0.01 using t-test. **D**, Expression of Notch1 proteins by Western blot in the myocardium of WT (n=4) and cKO (n=4) male mice 1-3 days after MI. For infarcted hearts either the intraventricular septum or free LV wall was employed. Data are expressed as fold changes relative to WT and presented as median and interquartile ranges with dot plots. *P<0.05 using Mann-Whitney rank sum test.

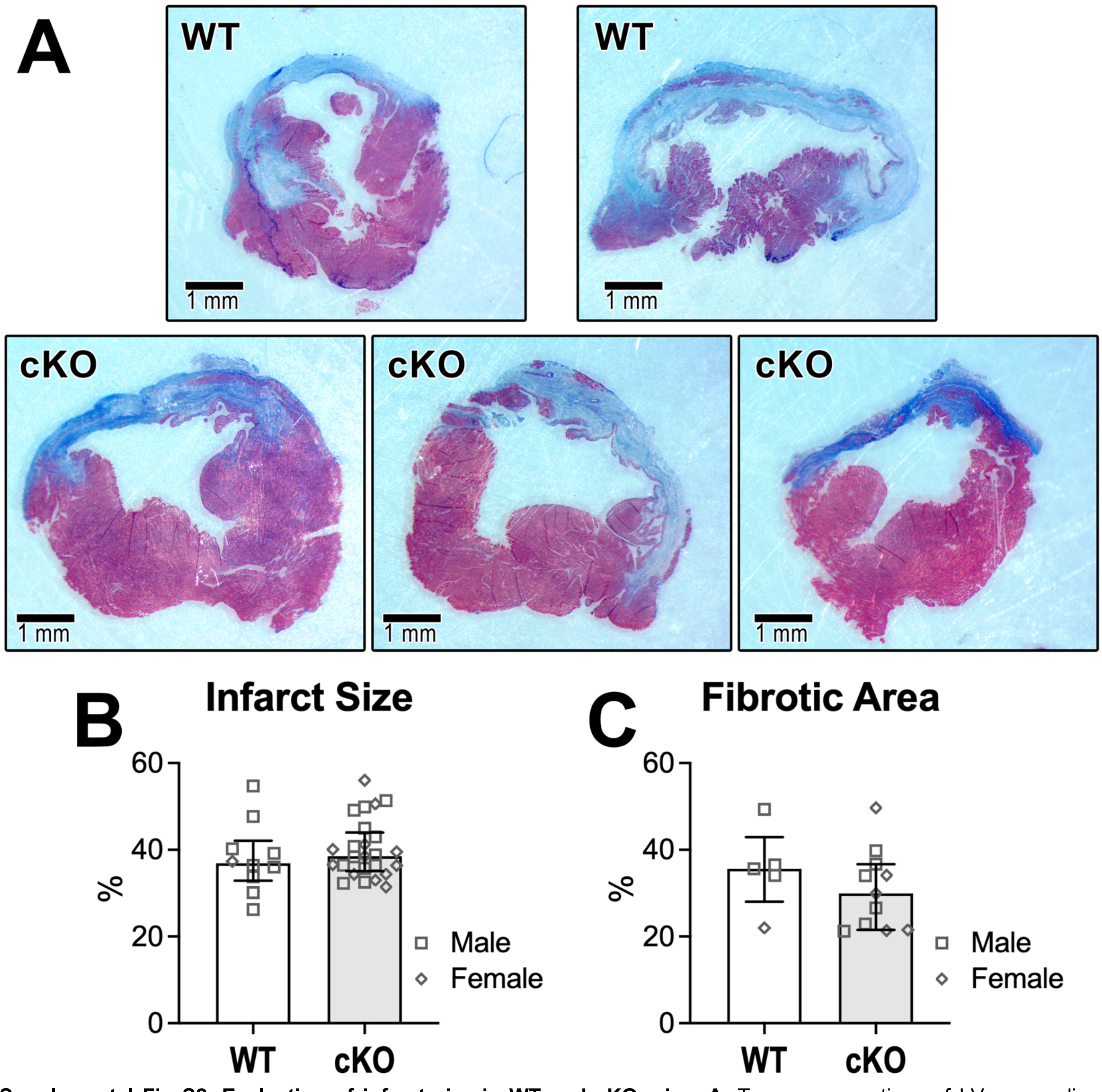

Supplemental Fig S2. Evaluation of infarct size in WT and cKO mice. A, Transverse sections of LV myocardium obtained from hearts of WT and cKO mice at ~1 month after coronary artery ligation. Fibrotic tissue is identified by trichrome staining (blue). **B,** Quantitative data for infarct size in formalin-fixed transverse LV sections at the mid-ventricular level from WT (n = 10, 9 males and 1 female) and cKO (n = 25, 13 males and 12 females) mice at 29-38 days after coronary artery ligation. Square and diamond symbols identify data for male and female mice, respectively. **C,** Quantification of the fibrotic tissue area, based on trichrome staining, expressed relative to the entire LV section in WT (n = 5, 4 males and 1 female) and cKO (n = 11, 6 males and 5 females) mice. Square and diamond symbols identify data for male and female mice, respectively.

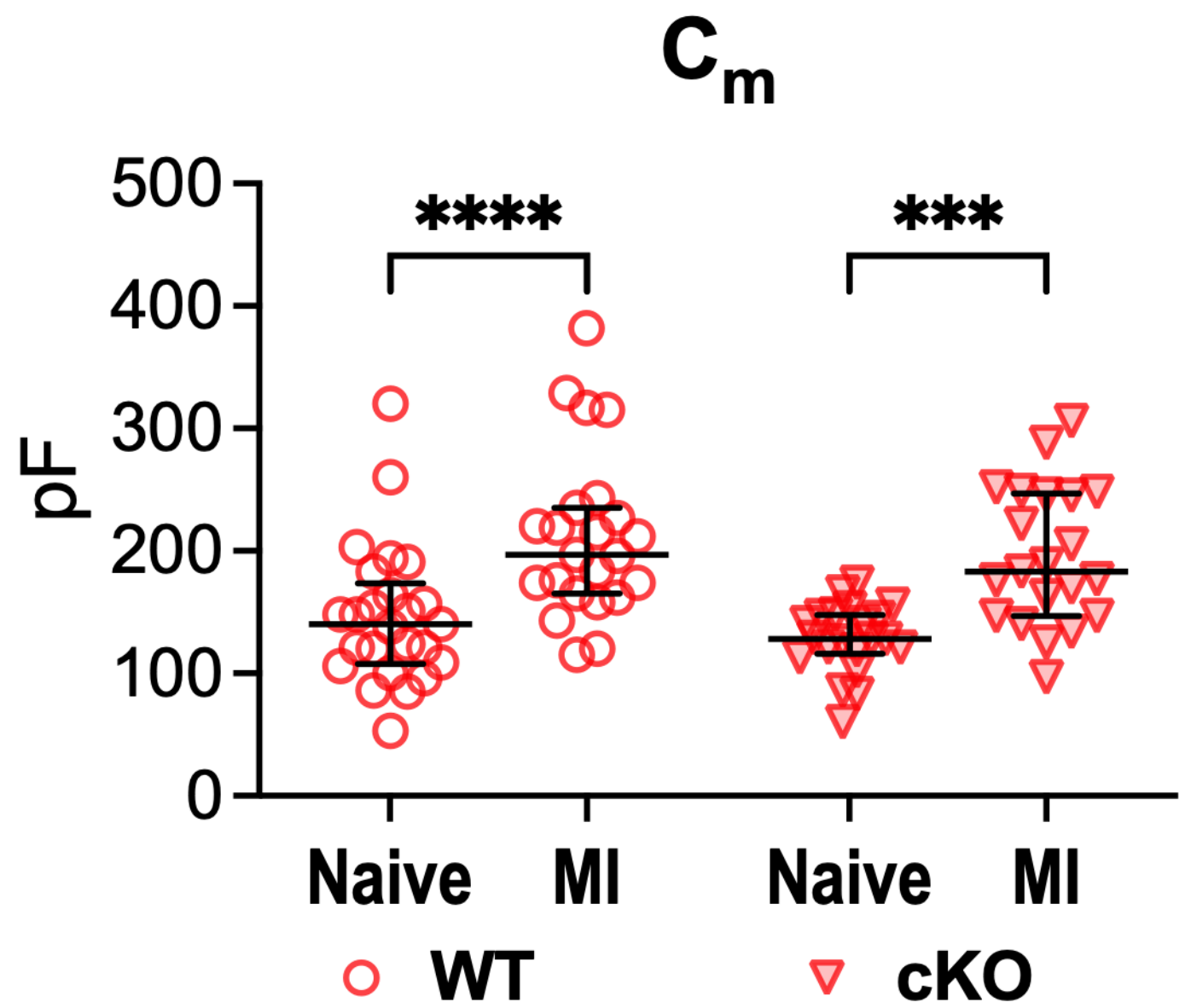

Supplemental Fig S3. Membrane capacitance of myocytes from naïve and infarcted female mice. Quantitative data for membrane capacitance (C_m) of cardiomyocytes used for the quantification of Kv currents (see Results section). Data are median and interquartile ranges with dot plots. ***$P<0.001$, ****$P<0.0001$ using two-way ANOVA followed by uncorrected Fisher's LSD test for multiple comparisons.

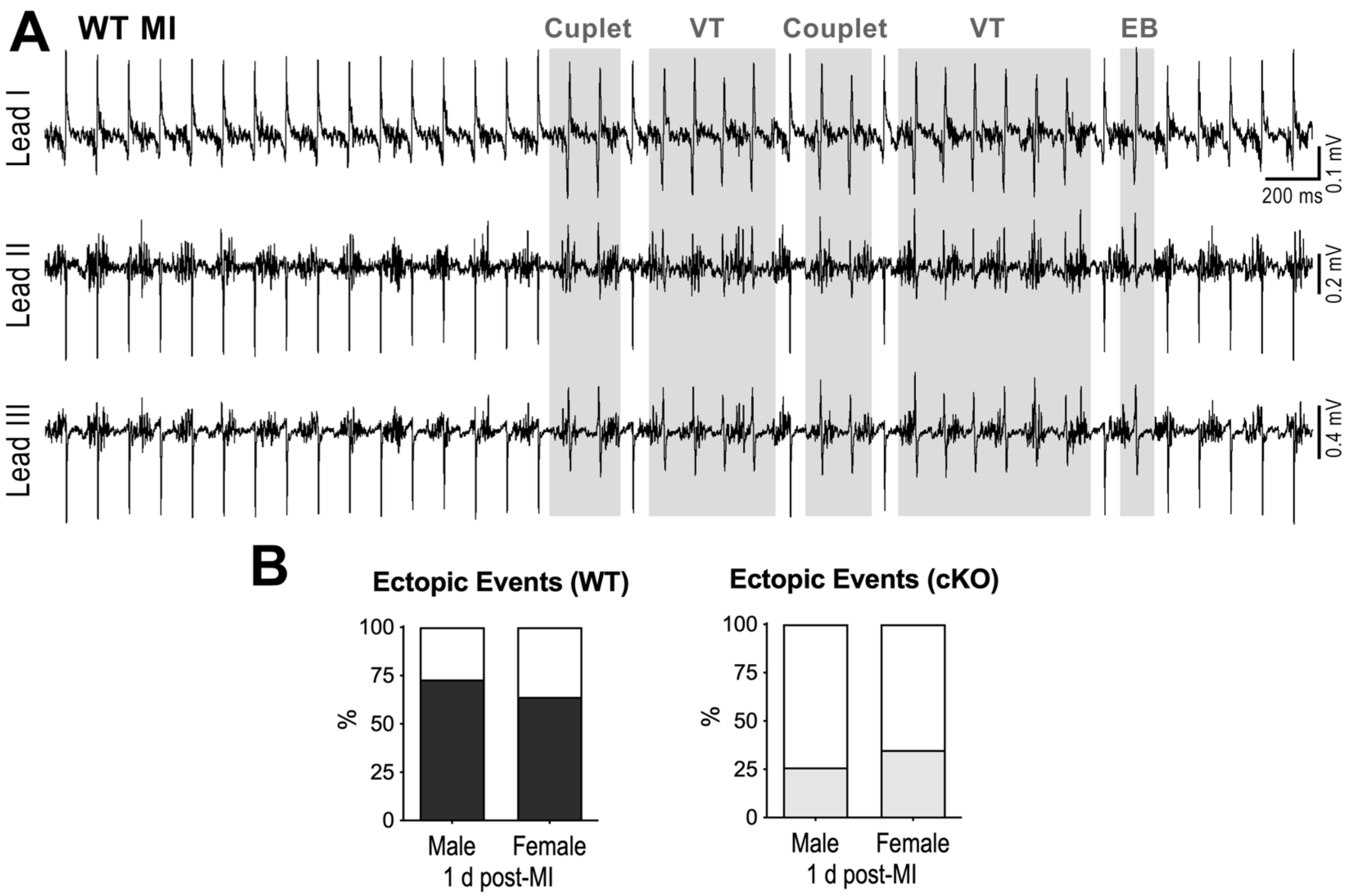

Supplemental Fig S4. Loss of *Notch1* gene and electrical disturbances following MI. A, ECG traces (lead I, II, and III) collected in a conscious WT female mouse at 1 day after MI. Gray areas identify ventricular ectopic beats (EB), couplets, and ventricular tachycardia (VT). **B**, Quantitative data for the fraction of male and female mice presenting ectopic events (solid segment) at 1 day after MI for WT (n=15, 11, respectively) and cKO (n= 19, 23, respectively) mice. Data are expressed as percentage of animals presenting ectopic events.

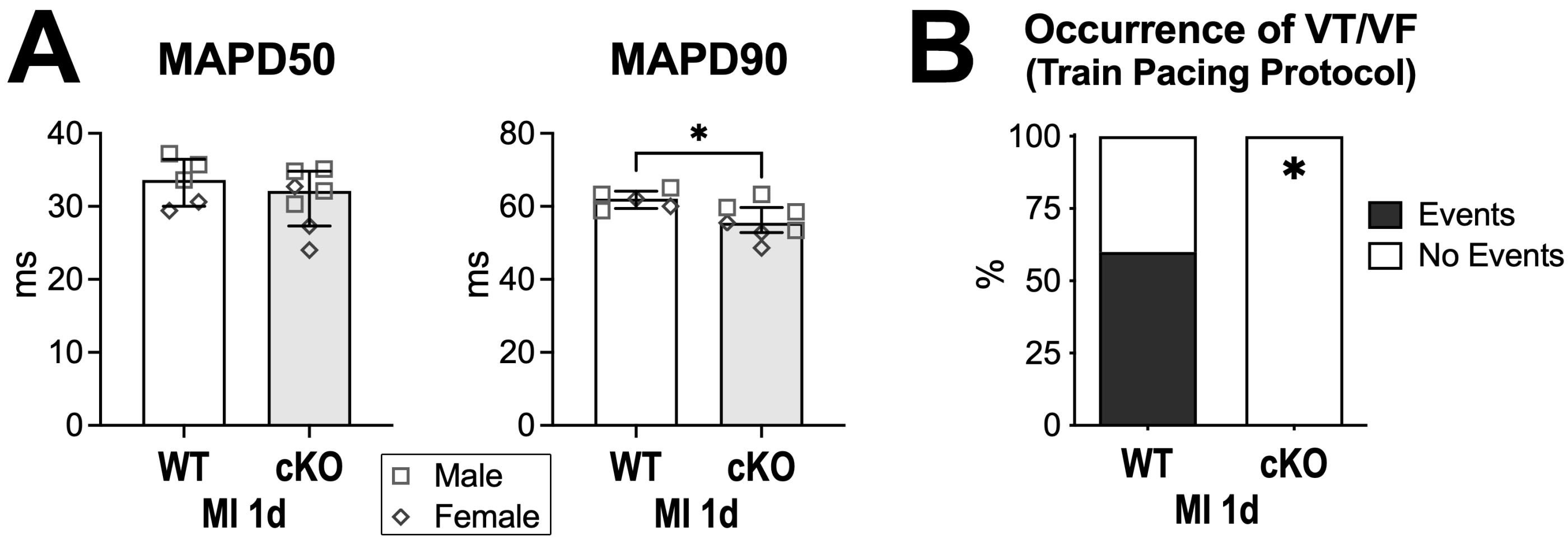

Supplemental Fig S5. Loss of *Notch1* gene attenuates electrical disturbance of the LV myocardium acutely after MI. A, Data for the duration of epicardial monophasic AP at 50% (MAP50) and 90% (MAP90) repolarization in the remote LV myocardium of hearts from WT (n=5; 3 males and 2 females) and cKO (n=7; 4 males and 3 females) mice at 1 day after MI. Data are median and interquartile ranges with dot plots. Square and diamond symbols identify data for male and female mice, respectively. *P<0.05 using t-test. **B,** Quantitative data for the fraction of hearts presenting VT/VF (solid segment) by incremental pacing. Hearts obtained from infarcted WT (n=5; 3 males and 2 females) and cKO (n=7; 4 males and 3 females) mice. Data are expressed as percentage of hearts presenting VT/VF. *P<0.05 vs WT using Fisher's exact test.

Table S1. Primers for PCR analysis

Gene	Primers
Mouse Hprt	F:5'- AGCCCCAAAATGGTTAAGGT -3'
	R: 5'- CAAGGGCATATCCAACAACA -3'
Mouse *Kcnip2*	F: 5'- TGAGGGTCTGGAACAACTCC-3'
	R: 5'- GGGACATTCGTTCTTGAAGC-3'
Mouse *Kcnd2*	F: 5'- TCAGCAAGCAAGTTCACCAG-3'
	R: 5'- TTCCCTGCTATGGTTTTTGG-3'
Mouse *Kcna5*	F: 5'- ATGAGGCCCATCACTGTAGG-3'
	R: 5'- AAAATTGGAGACGATGACGG-3'

Notch1

Chemiluminescence

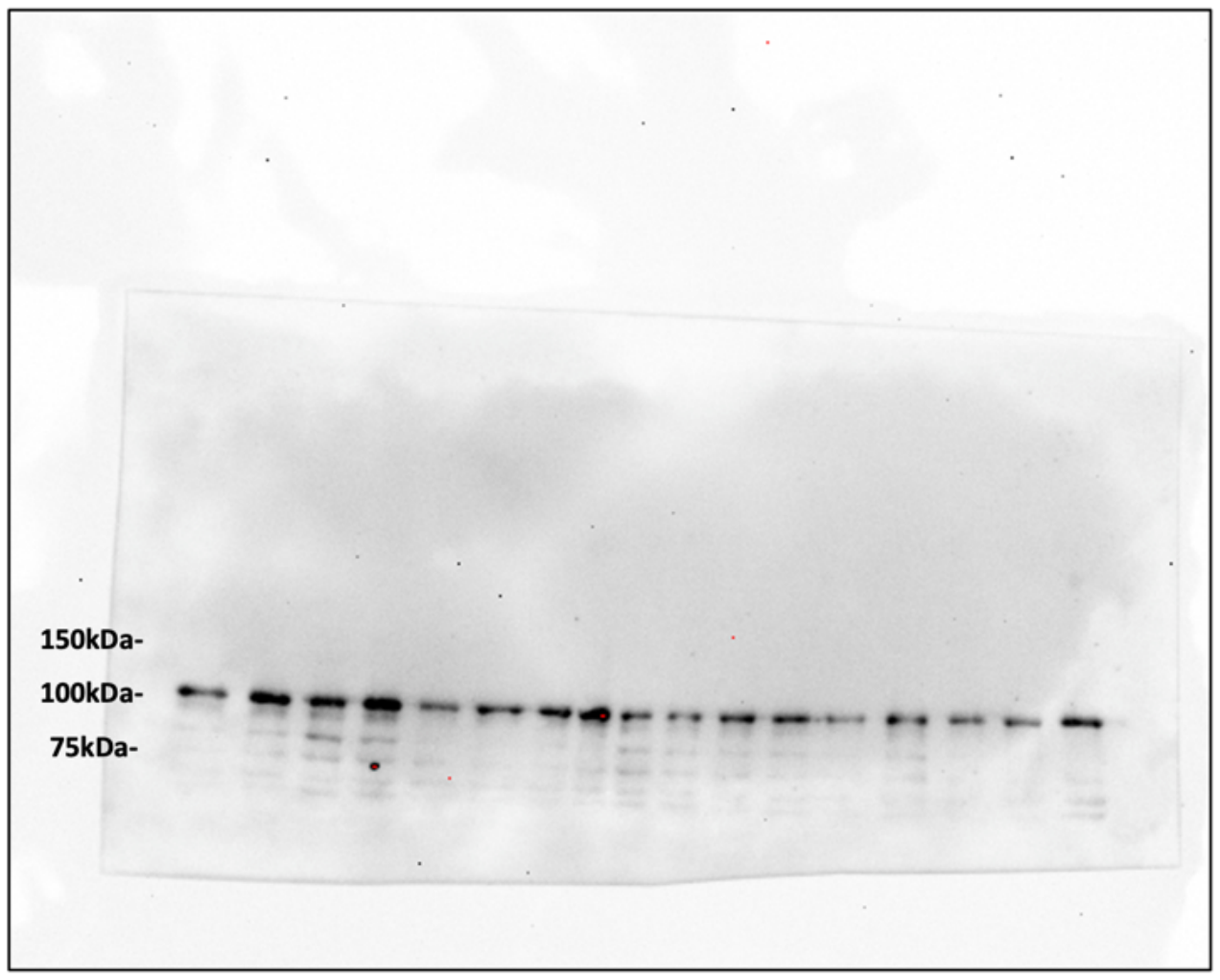

Brightfield

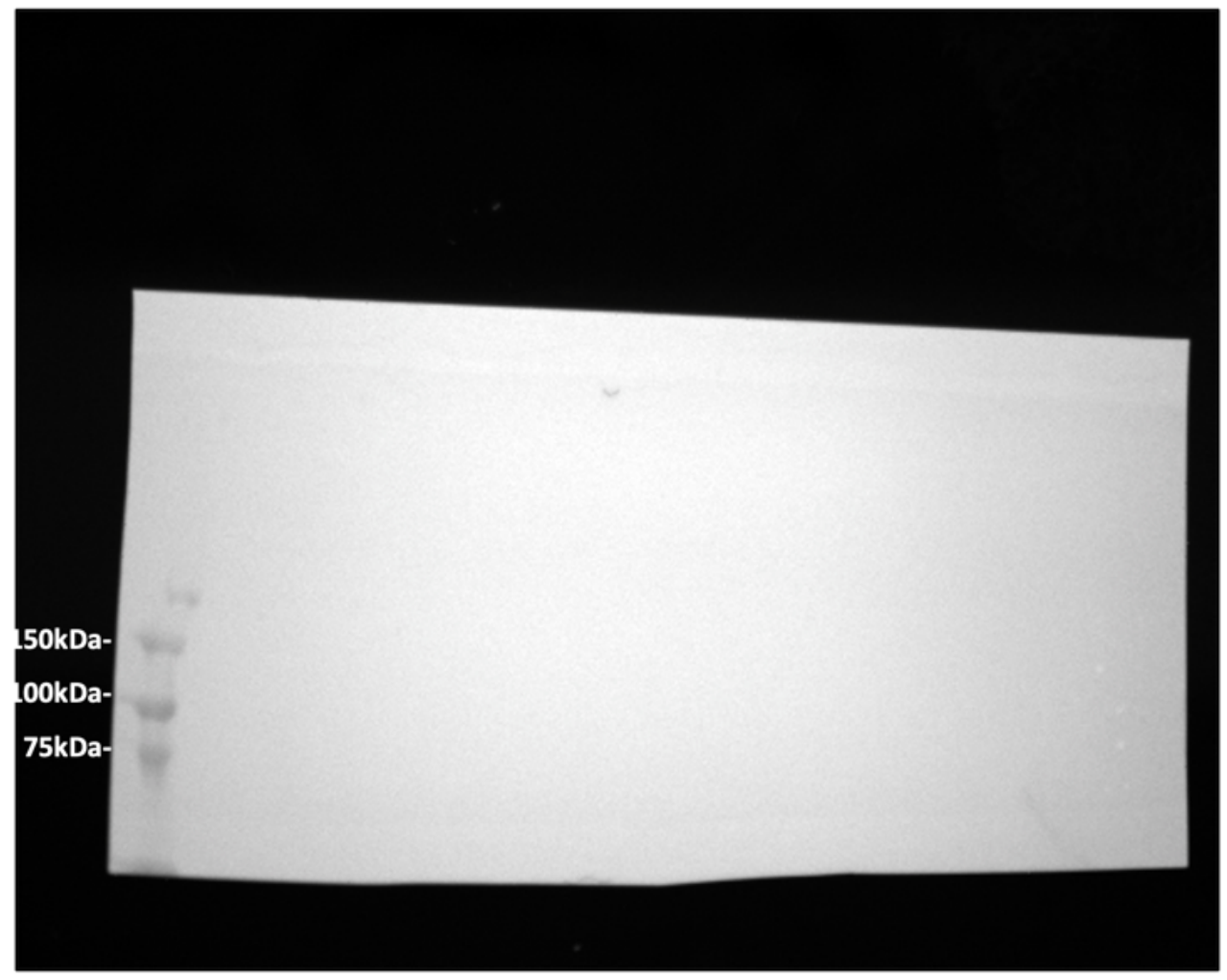

GAPDH

Chemiluminescence

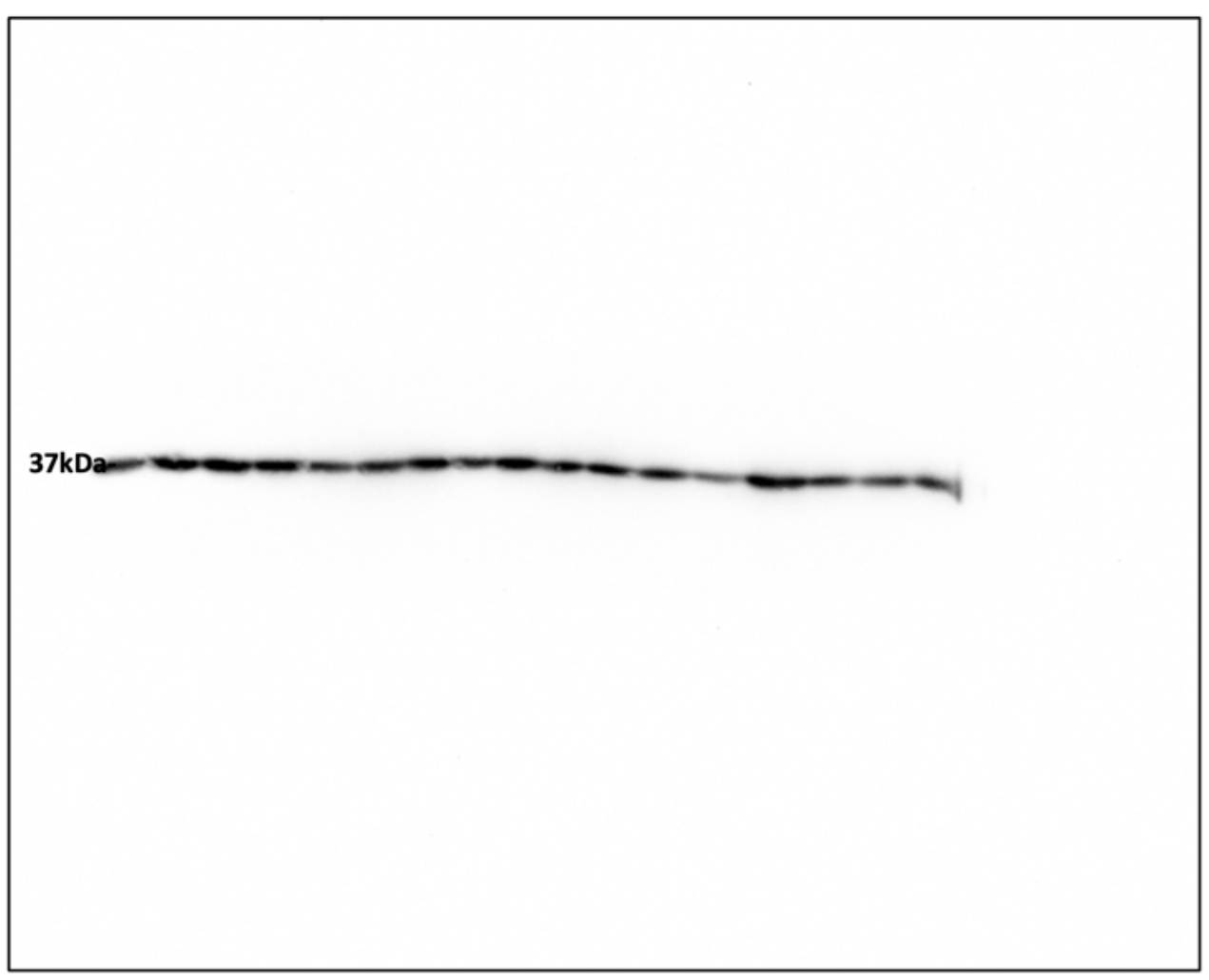

Brightfield

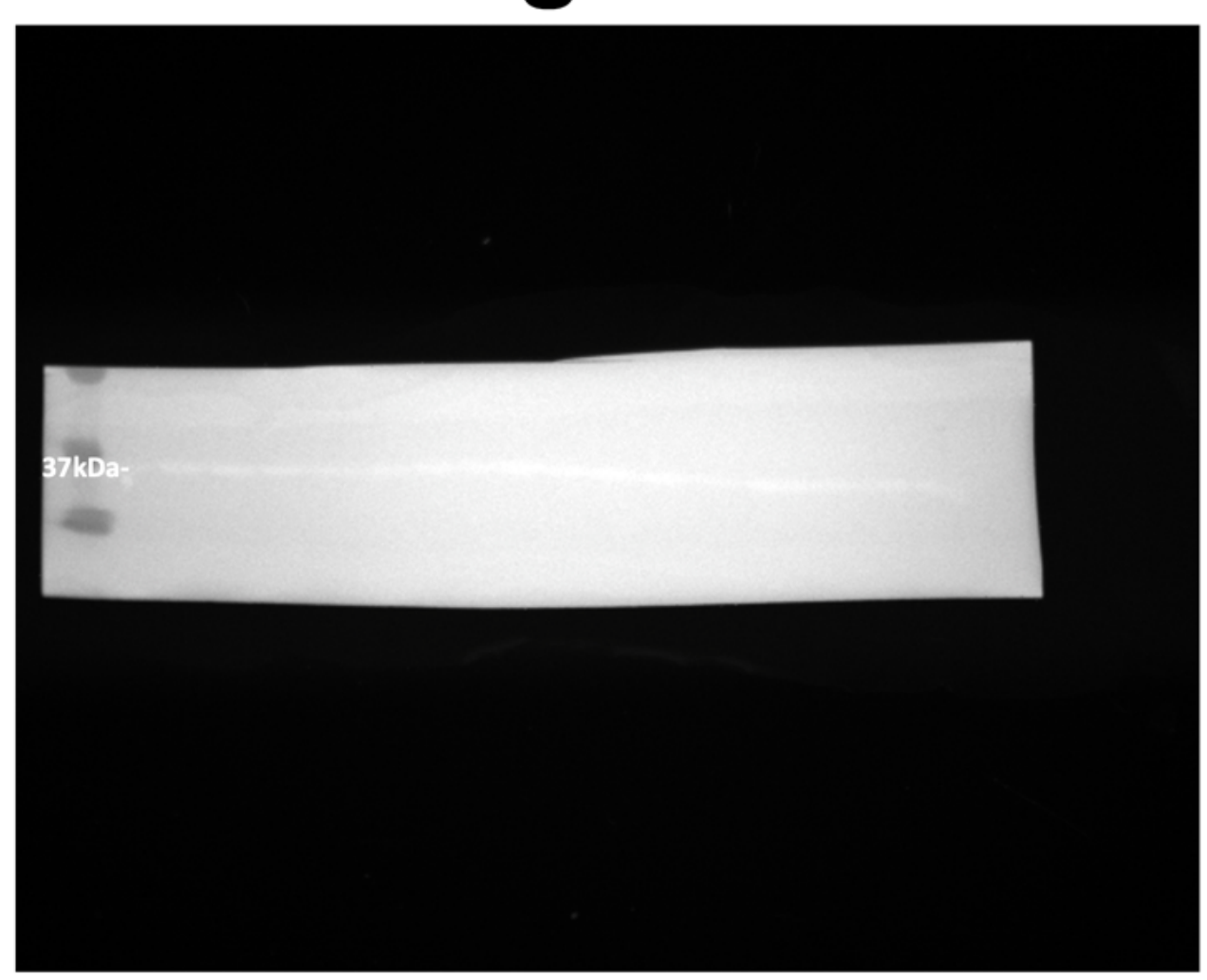

Uncropped Gel for Supplemental Fig S1B.

KChIP2

Chemiluminescence **Brightfield**

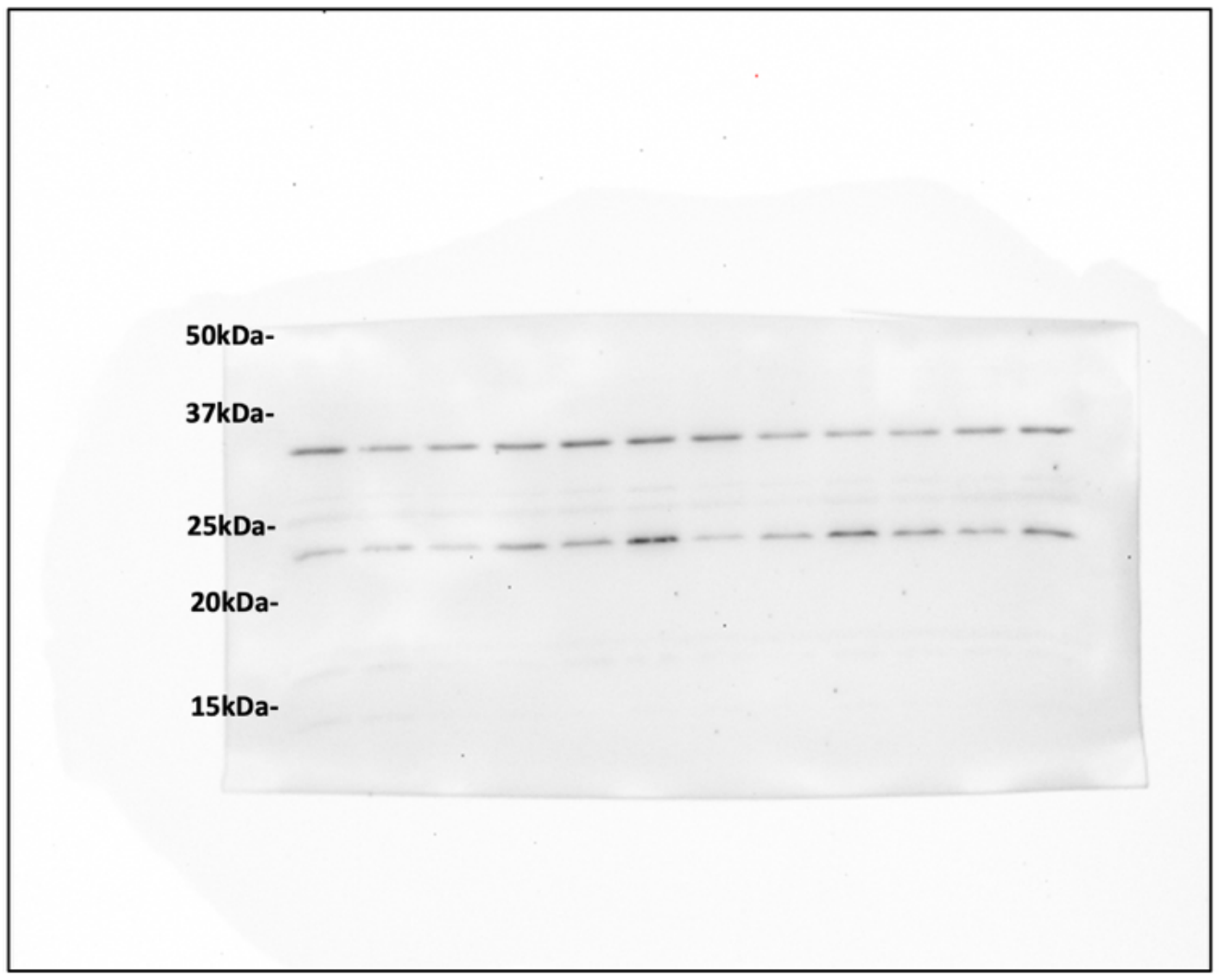

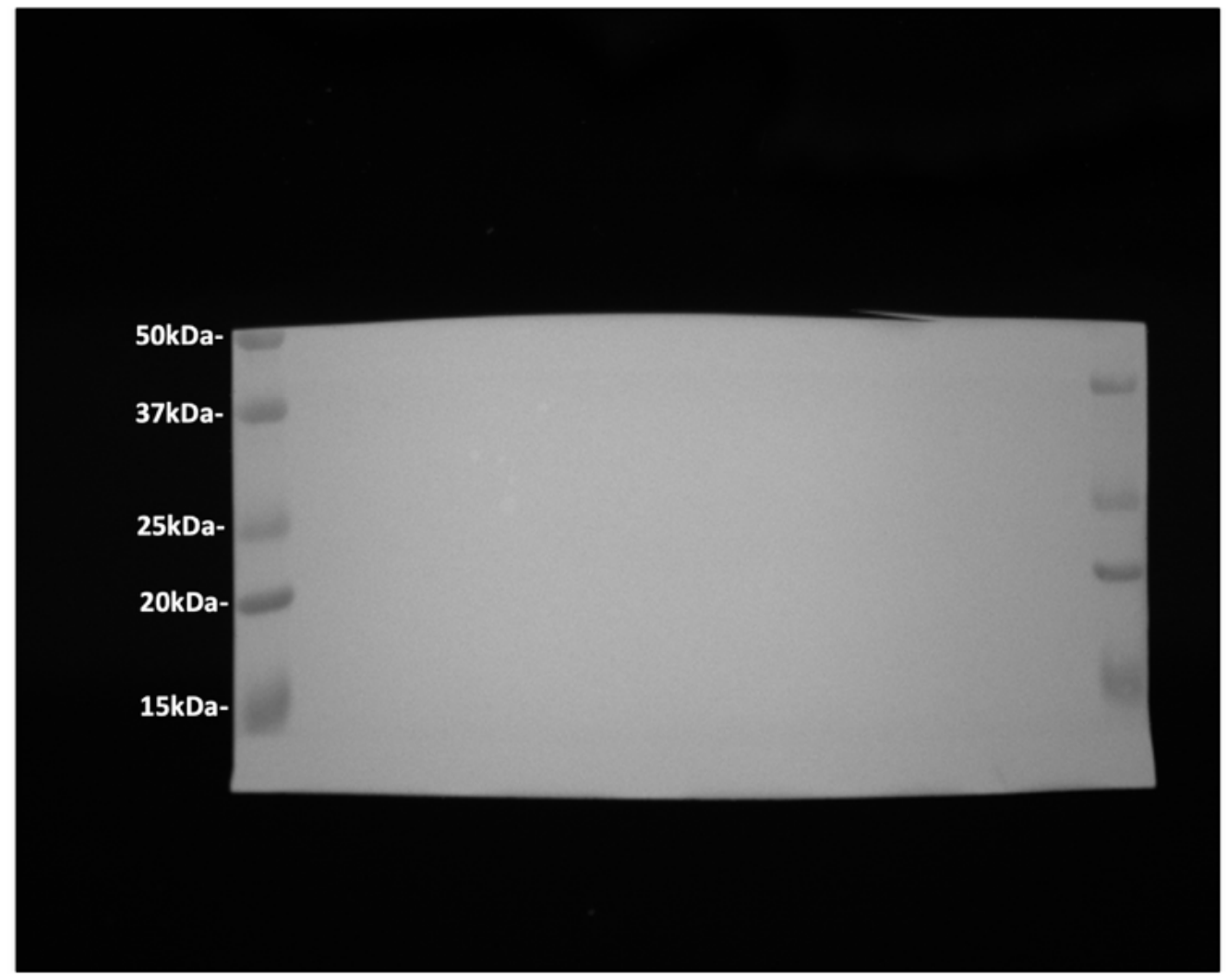

Vinculin

Chemiluminescence **Brightfield**

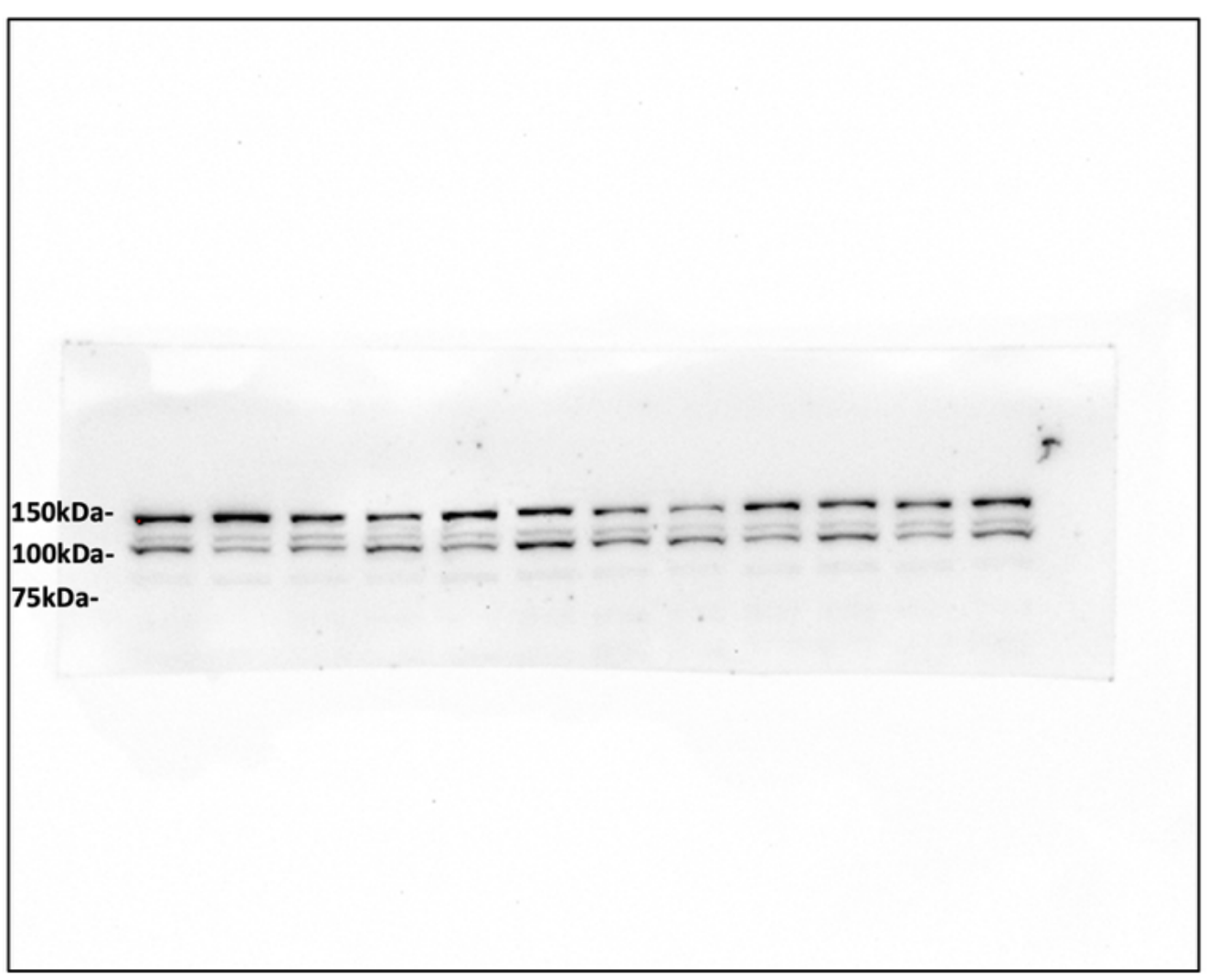

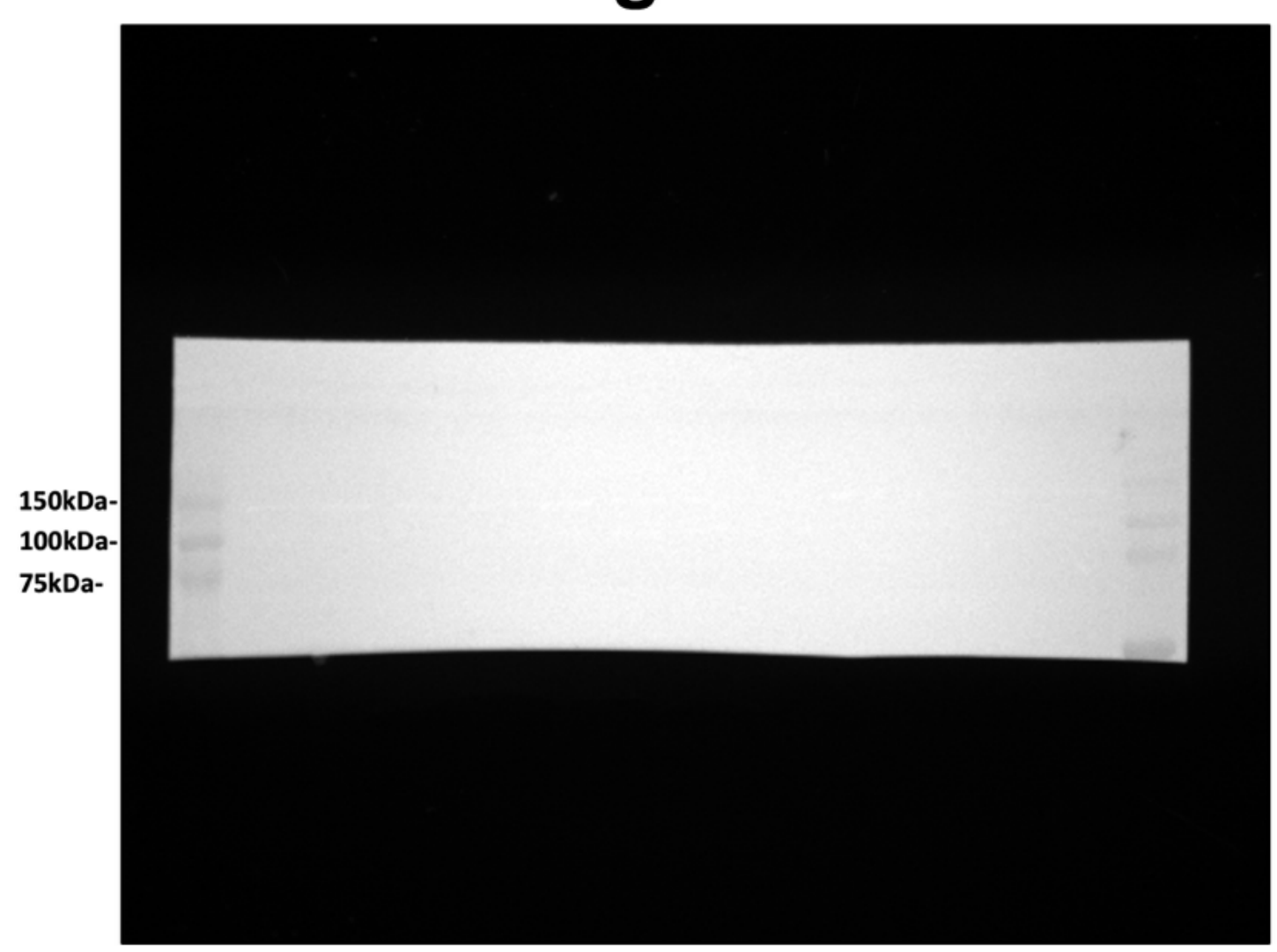

Uncropped Gel for Fig 5B.
